# Aberrant Chitinase 3‐Like 1 Expression in Basal Cells Contributes to Systemic Sclerosis Fibrosis

**DOI:** 10.1002/advs.202310169

**Published:** 2024-12-17

**Authors:** Xiuyuan Wang, Tianbao Ye, Junxia Huang, Feifei Hu, Chengjie Huang, Bei Gu, Xinzhi Xu, Ji Yang

**Affiliations:** ^1^ Department of Dermatology Zhongshan Hospital of Fudan University Shanghai 200032 China; ^2^ Sixth People's Hospital affiliated to Shanghai Jiao Tong University Shanghai 200233 China; ^3^ Xiamen Cardiovascular Hospital of Xiamen University School of Medicine Xiamen University Xiamen Fujian 361008 China; ^4^ State Key Laboratory of Oncogenes and Related Genes Institute for Personalized Medicine School of Biomedical Engineering Shanghai Jiao Tong University Shanghai 200030 China; ^5^ Shanghai Normal University Shanghai 200233 China

**Keywords:** chitinase 3‐like 1, fibrosis, interleukin‐17 receptor A, single‐cell RNA sequencing, systemic sclerosis

## Abstract

Systemic sclerosis (SSc) is an autoimmune disease characterized by extensive skin and internal organ fibrosis. However, the mechanism underlying fibrosis remains unclear, and effective treatments for halting or reversing fibrosis are lacking. In this study, single‐cell RNA sequencing is used to obtain a comprehensive overview of skin cells from patients with SSc and healthy controls. A subset of basal cells with high chitinase 3‐like 1 (Chi3L1) expression, which potentially plays an important role in fibroblast activation, is identified in SSc. Subsequently, patients with SSc are present with increased expression of Chi3L1 in the skin and serum, and elevated serum levels are associated with skin induration and pulmonary function. Furthermore, Chi3L1 promoted the differentiation of SSc dermal fibroblasts into myofibroblasts, and Chi3L1‐deficient (Chi3L1‐/‐) mice showed amelioration of fibrosis in a bleomycin‐induced SSc (BLM‐SSc) model. Mechanistically, Chi3L1 mediates fibroblast activation primarily by interacting with interleukin‐17 receptor A (IL‐17RA), thereby initiating downstream nuclear factor kappa B and mitogen‐activated protein kinases signaling pathways. Moreover, the anti‐fibrotic effect of IL‐17RA antagonists in BLM‐SSc mice is demonstrated. In conclusion, Chi3L1 is a potential biomarker for the degree of fibrosis in SSc. Chi3L1 and its receptor, IL‐17RA, are promising therapeutic targets for patients with SSc.

## Graphical abstract

This study identifies a cluster of *Chi3L1^hi^* basal cells enriched in patients with SSc. Chi3L1 can directly bind to IL‐17RA on fibroblasts, and then activate the downstream NF‐kB and MAPK pathways, which promotes fibroblast activation and collagen deposition. Chi3L1 deficiency and anti‐IL‐17RA monoclonal antibodies treatment alleviated fibrosis of bleomycin‐induced SSc mice. These findings offer insights into potential SSc therapies.

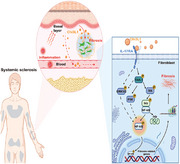

## 1 Introduction

Systemic sclerosis (SSc) is an autoimmune connective tissue disease that causes extensive skin and internal organ fibrosis.^[^ ^1^ ^]^ At present, SSc is primarily classified into two forms: limited cutaneous SSc (lcSSc) and diffuse cutaneous SSc (dcSSc), which are distinguished by the extent of skin involvement.^[^ ^2^, ^3^ ^]^ Despite its relatively low prevalence, SSc has the highest mortality rate among all rheumatic diseases.^[^ ^4^ ^]^ Similar to other fibrotic diseases, a key element in the pathogenesis of fibrosis in SSc is the transformation of fibroblasts into myofibroblasts expressing α‐smooth muscle actin (α‐SMA), leading to excessive collagen synthesis and immoderate extracellular matrix (ECM) deposition.^[^ ^5^, ^6^, ^7^ ^]^ However, the specific mechanisms underlying fibroblast activation in SSc have not been fully elucidated, and effective anti‐fibrotic strategies are still lacking.

Keratinocytes, the primary cellular component of the epidermis, have long been overlooked in the pathogenesis of dermal fibrosis.^[^ ^8^ ^]^ Recent research, however, has suggested that the homeostasis regulating the interaction between dermal and epidermal cells is disrupted in SSc.^[^ ^9^, ^10^, ^11^ ^]^ Keratinocytes may play an important role in fibroblast activation and fibrosis. Previous studies have shown that conditioned media from SSc keratinocytes can upregulate the expression of collagen type I (Col1A1) and α‐SMA in fibroblasts compared with that in media from healthy donors.^[^ ^12^ ^]^ Notably, this effect can be independent of transforming growth factor‐β (TGF‐β) and accompanied by an imbalance in the nuclear factor kappa B (NF‐kB) pathway.^[^ ^12^ ^]^ Nevertheless, the key mediators and mechanisms by which keratinocytes promote fibroblast activation remain unclear.

Chitinase‐like proteins (CLPs), which belong to glycoside hydrolase family 18 but are distinct from true chitinases, are non‐enzymatic proteins expressed in various organs of the human body.^[^ ^13^ ^]^ Chitinase 3‐like 1 (Chi3L1), also known as breast regression protein 39 in mice and human homolog YKL‐40, is a member of the CLP family and is secreted by various immune and structural cells.^[^ ^14^, ^15^ ^]^ Extensive studies have indicated that Chi3L1 plays a crucial role in various inflammatory reactions, tissue remodeling responses, and fibrotic processes, such as atopic dermatitis, asthma, lung fibrosis, and liver fibrosis.^[^ ^16^, ^17^, ^18^, ^19^ ^]^ Moreover, evidence demonstrates that skin cells from patients with SSc secrete Chi3L1, and these patients exhibit higher serum Chi3L1 concentrations.^[^ ^20^, ^21^ ^]^ However, the main source of Chi3L1 and its potential roles and underlying mechanisms in SSc remain elusive.

In the present study, the single‐cell RNA sequencing (scRNA‐seq) of the digested total skin cells from patients with SSc and healthy control (HC) participants was employed to explore the pathogenesis of fibrosis in SSc. We identified *Chi3L1^hi^* basal cells as a potential key cell population involved in crosstalk with fibroblasts, especially inactive fibroblasts. Furthermore, we presented the solides evidence indicating that Chi3L1, as a critical protein predominantly expressed by basal cells in the skin, is actively engaged in fibroblast activation through NF‐kB and mitogen‐activated protein kinase (MAPK) pathway by interacting with the interleukin‐17 receptor A (IL‐17RA) of fibroblasts in SSc. Our study highlights a novel role of Chi3L1 in promoting fibrosis in SSc, and our findings suggest the potential anti‐fibrotic capability and clinical applicability of IL‐17RA antagonists in patients with SSc.

## 2 Results

### 2.1 ScRNA‐Seq Reveals *Chi3L1^hi^* Basal Cells as a Key Cell Population Participating in Fibroblast Activation in SSc

**Figure 1:**
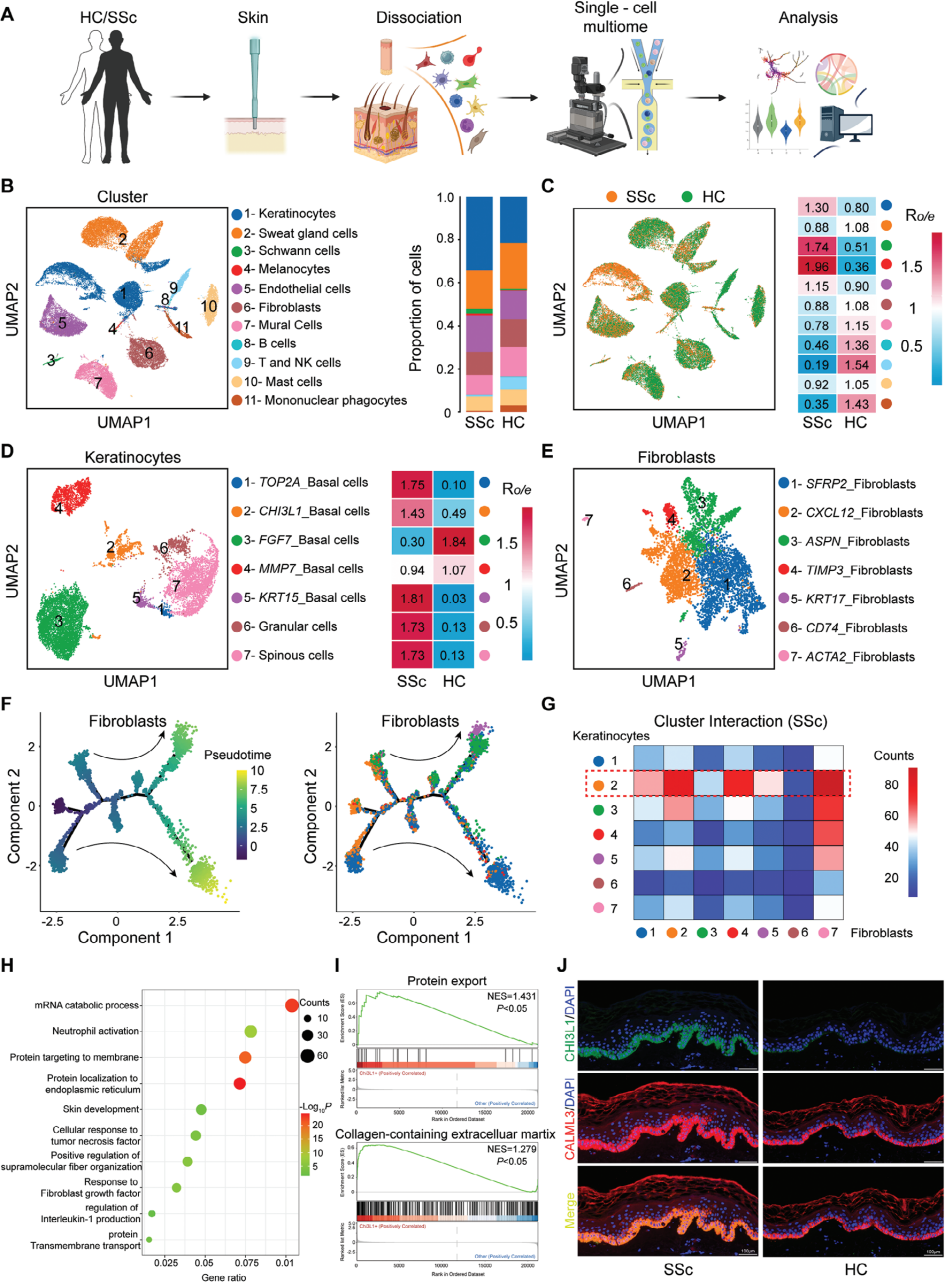
ScRNA‐seq reveals *Chi3L1^hi^* basal cells as a key cell population participating in fibroblast activation in SSc. A) Schematic of scRNA‐seq for skin biopsies. B) The UMAP of cell subpopulation (left) and proportion of cell clusters (right). C) Cell distribution distinguished between SSc and HC (left) and enrichment preferences of cell clusters expressed (right). D) The UMAP of keratinocytes subpopulation (left) and enrichment preferences (right). E) The UMAP of fibroblasts subpopulation. F) Pseudotime analysis on fibroblasts, showing by pseudotime (left), or by fibroblast cluster (right). G) Heatmap of interaction strength between keratinocyte and fibroblast clusters. H) BP terms of DEGs. I) GSEA analysis via GO and KEGG database. J) Representative double immunofluorescent images of skin stained with indicated antibodies.

Previous studies have shown that the transformation of fibroblasts into myofibroblasts is an important factor in the pathogenesis of fibrosis in SSc.^[^ ^22^ ^]^ To gain deeper insights, we performed scRNA‐seq on the skin of three patients with SSc and three HCs (**Figure** **1**A). The transcriptomes of 44571 cells (SSc:17797, HC:26774) were obtained, and uniform manifold approximation and projection (UMAP) identified 11 cell clusters (Figure 1B). Cell proportion and enrichment preferences indicated that keratinocytes accounted for the largest proportion of the cell population and were remarkably enriched in SSc (Figure 1B,C).^[^ ^23^ ^]^ Previous studies have indicated that keratinocytes are involved in fibroblast activation.^[^ ^8^ ^]^ Therefore, to further investigate keratinocytes, we subdivided them and revealed that *Chi3L1^hi^*, *TOP2A^hi^*, and *KRT15^hi^* basal cells and granular and spinous cells were enriched in SSc (Figure 1D).

Fibroblasts, crucial target cells in fibrosis, were also subdivided and extensively investigated (Figure 1E). To determine the lineage relationships and corresponding gene expression, pseudotime analysis of fibroblasts was performed. Most *CXCL12^hi^* fibroblasts were detected at the starting point of the trajectory (Figure 1F). The expression of *ACTA2*, also known as *α‐SMA*, and *Periostin* (*Postn*), previously defined as a myofibroblast marker, was observed in the mid to late stages of differentiation, which is consistent with the understanding of fibroblasts differentiating into myofibroblasts in SSc (Figure S1A, Supporting Information).^[^ ^24^ ^]^ Moreover, in fibroblast characteristic scoring, *CXCL12^hi^* fibroblasts demonstrated relatively low collagen synthesis and proliferative capacity, lacking features typical of myofibroblasts (Figure S1B, Supporting Information), further suggesting that *CXCL12^hi^* fibroblasts tended to be inactive.

To explore the key keratinocyte clusters involved in fibroblast activation, interaction strength analysis between keratinocytes and fibroblast clusters was conducted (Figure 1G). *Chi3L1^hi^* basal cells demonstrated the strongest interaction with fibroblasts, particularly *CXCL12^hi^* fibroblasts. To further elucidate the specificity of *Chi3L1^hi^* basal cells relative to that of the other basal cells, gene ontology (GO) analysis was conducted using differentially expressed genes (DEGs). Biological process (BP), cell component (CC), and molecular function (MF) term analyses delineated the functions and characteristics of *Chi3L1^hi^* basal cells, summarily involving active protein synthesis and secretion, participation in collagen‐containing ECM generation, regulation by inflammatory responses, and involvement in skin development (Figure 1H; Figure S2A, Supporting Information). Furthermore, gene set enrichment analysis (GSEA) based on GO and Kyoto Encyclopedia of Genes and Genomes (KEGG) analyses of the DEGs substantiated this conclusion (Figure 1I; Figure S2B, Supporting Information). Double immunofluorescence staining of Chi3L1 and CALML3, another marker of *Chi3L1^hi^* basal cells, validated the enrichment of *Chi3L1^hi^* basal cells in the skin of patients with SSc, as indicated by the transcriptomic data (Figure 1J; Figure S3, Supporting Information). More importantly, considering both expression levels and the proportion of expressing cells, *Chi3L1* was predominantly expressed in basal cells compared with other cell types in the skin (Figure S4, Supporting Information).

Taken together, these findings present that *Chi3L1^hi^* basal cells are enriched in SSc and play a pivotal role in the crosstalk with fibroblasts, particularly with fibroblasts primed to differentiate into myofibroblasts. Moreover, as a secreted protein involved in various fibrotic diseases, Chi3L1 stood out as a potentially crucial molecule in fibroblast activation in SSc.^[^ ^16^ ^]^

### 2.2 Chi3L1 is Overexpressed in SSc Skin and Serum, and Serum Levels Correlate with Fibrosis in SSc

**Figure 2:**
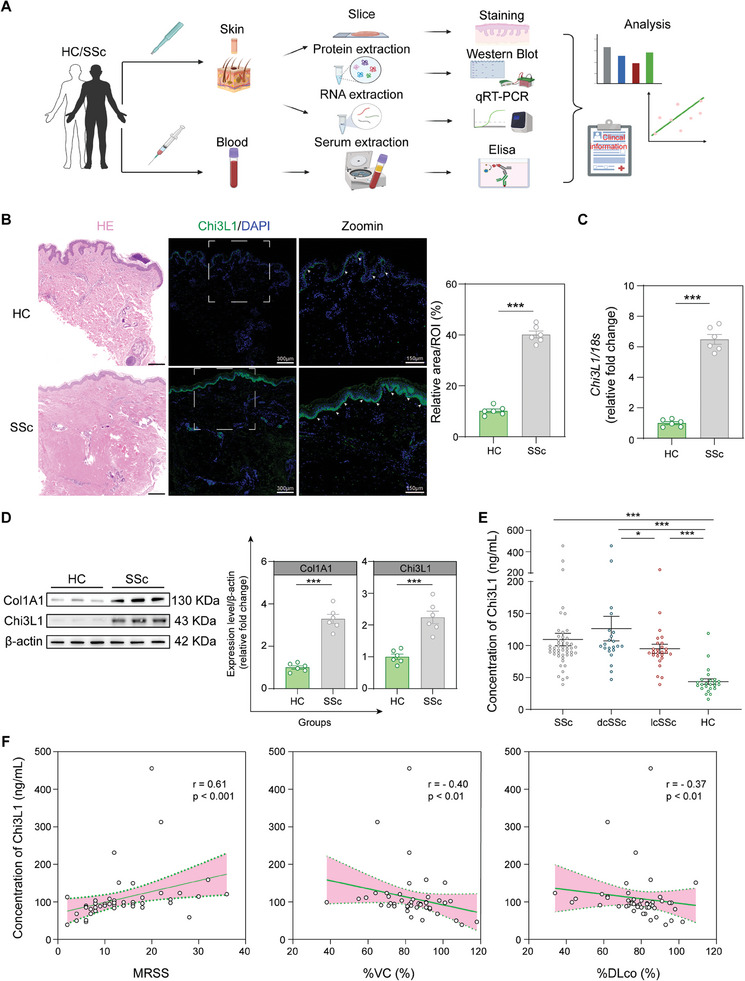
Chi3L1 is overexpressed in SSc skin and serum, and serum levels correlate with fibrosis in SSc. A) Diagram of the experimental approach. B) Representative HE and immunofluorescence staining images with relative quantification in the skin of patients with SSc and HCs, ROI, region of interest. *n* = 6/ea. C) Relative mRNA expression of *Chi3L1* in the skin of SSc patients and HC, *n* = 6/ea. D) Representative WB and relative quantification of Col1A1 and Chi3L1 in the skin of patients with SSc and HCs, *n* = 6/ea. E) Serum Chi3L1 levels of patients with SSc and HCs. F) Correlations between serum Chi3L1 levels and the following parameters in patients with SSc (*n* = 48): MRSS, %VC, %DLco. Data are presented as mean ± SEM. B, C, and D, by unpaired Student's *t*‐test. E, by two‐tailed Mann–Whitney U test. F, by Spearman's rank correlation test. *p < 0.05, *** *p* < 0.001 compared with corresponding groups.

To delve into the role of Chi3L1 in SSc, we assessed its expression in the skin and serum of patients with SSc and HCs. Additionally, we assessed the correlation between serum Chi3L1 levels, the modified Rodnan skin score (MRSS), and pulmonary function (**Figure** **2**A).^[^ ^25^ ^]^ Hematoxylin‐eosin (HE) staining indicated exaggerated dermal fibrosis in patients with SSc, while immunofluorescence staining revealed the upregulation of Chi3L1 in the epidermis and dermis, especially in the basal layer (Figure 2B). Furthermore, real‐time polymerase chain reaction (qPCR) analysis and western blotting (WB) confirmed elevated Chi3L1 expression levels in the skin of patients with SSc, with highly expressing of Col1A1 simultaneously (Figure 2C,D).

Next, we measured the Chi3L1 concentration in the serum of 48 patients with SSc (22dcSSc, 26lcSSc) and 24 HCs using enzyme‐linked immunosorbent assay (ELISA) (Table S1, Supporting Information). Serum Chi3L1 levels were significantly elevated in patients with SSc compared with those in HCs (Figure 2E). Notably, patients with dcSSc exhibited higher circulating Chi3L1 levels than those in patients with lcSSc. Additionally, serum Chi3L1 levels were found to positively correlate with MRSS, while exhibiting a negative correlation with the percentage predicted values of vital capacity (%VC) and diffusing capacity for carbon monoxide (%DLco) (Figure 2F).

These findings demonstrated the high expression of Chi3L1 in both the skin and serum of patients with SSc. Moreover, serum Chi3L1 levels correlated with fibrosis, serving as a potential biomarker of fibrosis in SSc.

### 2.3 Chi3L1 Promotes the Transformation of SSc Dermal Fibroblasts (DFs) to Myofibroblasts

**Figure 3:**
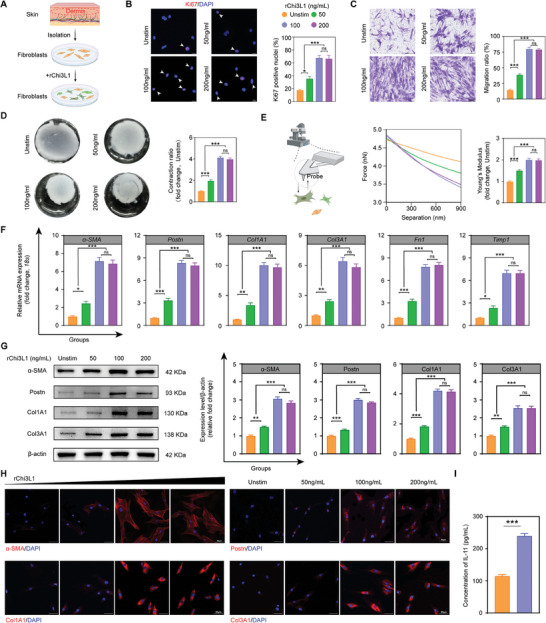
Chi3L1 promotes the transformation of SSc DFs to myofibroblasts. A) Schematic of the experimental protocol. B) Representative immunofluorescent images of proliferative cells with Ki67‐labeled nuclei. Quantification is shown on the right. *n* = 6/ea. C) Representative images and quantification of transwell assay at 24 h after stimulation with different concentrations of rChi3L1. *n* = 6/ea. D) Representative images and relative quantification of collagen gel contractility assay after 24 h of rChi3L1 administration. Quantification is shown on the right. *n* = 6/ea. E) Schematic of AFM for cell mechanical properties measurement (left). Representative force‐separation curve of DFs after stimulation (middle). Relative quantification of the cell Young's modulus in corresponding groups (right). *n* = 6/ea. F) Relative fold change of indicated mRNA expression level after stimulation. *n* = 6/ea. G) Representative WB and quantification of α‐SMA, Postn, Col1A1 and Col3A1 in cell lysates. *n* = 6/ea. H) Representative immunofluorescent micrographs of DFs stained with indicated antibodies after administration. I) IL‐11 secretion by SSc DFs stimulated by Chi3L1 (100 ng mL^−1^) or media alone for 24h. *n* = 6/ea. Unstim, unstimulated. Data are presented as mean ± SEM. B–G, by one‐way ANOVA followed by Bonferroni post hoc test. I, by unpaired Student's *t*‐test. ns = no significance, *p < 0.05,**p < 0.01, ***p < 0.001 compared with corresponding groups.

To further investigate the in vitro effects of Chi3L1, we stimulated DFs from SSc patients with recombinant Chi3L1 (rChi3L1) (**Figure** **3**A). The CCK‐8 assay indicated that cell proliferation significantly increased at concentrations starting from 50 ng mL^−1^, and no significant difference between 100 and 200 ng mL^−1^ was observed (Figure S5A, Supporting Information). In contrast, the LDH assay revealed no significant cytotoxicity at concentrations ranging from 0 to 200 ng mL^−1^; however, a marked increase in cytotoxicity was observed at 400 ng mL^−1^ (Figure S5B, Supporting Information). Therefore, a concentration range of 0–200 ng mL^−1^ rChi3L1 was selected for further experiments. The Ki67 immunofluorescence staining results further supported the findings from the CCK‐8 assay (Figure 3B). Concurrently, transwell and scratch assays demonstrated that the migratory capability of SSc DFs could be augmented by rChi3L1 (Figure 3C; Figure S5C, Supporting Information). Furthermore, SSc DFs displayed an enhanced capacity to contract the collagen gel matrix after rChi3L1 stimulation (Figure 3D).^[^ ^26^ ^]^ Atomic force microscopy (AFM) showed distinct force‐separation curves of SSc DFs at high rChi3L1 concentrations, demonstrating that rChi3L1 can increase the stiffness of SSc DFs (Figure 3E). This enhancement in stiffness may be associated with alterations in the cytoskeleton induced by changes in cellular actin levels.^[^ ^27^ ^]^ Collectively, the increased contractility and stiffness implied that Chi3L1 promoted the differentiation of SSc DFs into myofibroblasts.^[^ ^28^ ^]^ The qPCR assay further confirmed the rChi3L1‐induced upregulation of the expression of genes encoding ECM components (*Col1A1*, *collagen type III*, *Col3A1* and *fibronectin*, *FN1*), myofibroblast marker molecules (*α‐SMA* and *Postn*) and fibrosis‐associated enzymes (*tissue inhibitors of metalloproteinase 1*, *Timp1*) (Figure 3F). WB analysis and immunofluorescence images corroborated that α‐SMA, Postn, Col1A1, and Col3A1 were highly expressed in SSc DFs following rChi3L1 stimulation (Figure 3G,H; Figure S5D, Supporting Information). Additionally, stimulation of SSc DFs with rChi3L1 led to the increased secretion of interleukin‐11 (IL‐11), a classic pro‐fibrotic molecule autocrinely secreted by fibroblasts in SSc (Figure 3I).^[^ ^29^, ^30^, ^31^ ^]^ All these data validated the ability of Chi3L1 to effectively boost SSc DF activation.

### 2.4 Chi3L1 Knock‐out (KO) Alleviates Fibrosis in Bleomycin‐Induced SSc Model (BLM‐SSc) Mice

**Figure 4:**
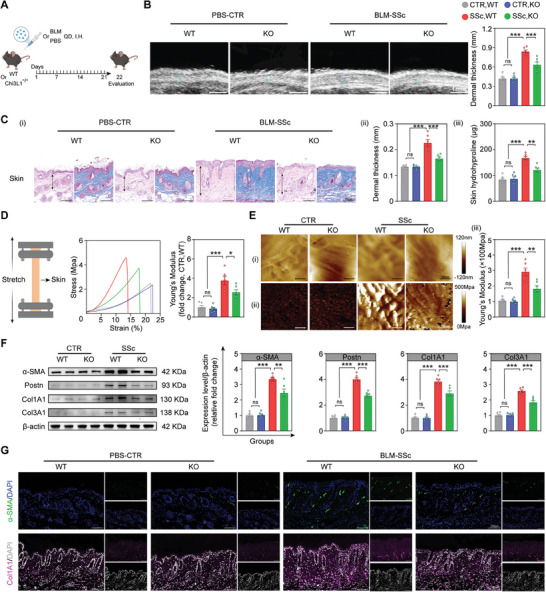
Chi3L1 KO alleviates fibrosis in BLM‐SSc mice. A) Diagram of the experimental protocol. QD, once a day. B) Representative skin ultrasound images of the corresponding groups. Quantification of dermal thickness is shown on the right. *n* = 6/ea. C) (i) Representative HE (left) and Masson trichrome (right) staining of skin in the corresponding groups. (ii) Quantification of dermal thickness. (iii) Hydroxyproline content of skin samples. *n* = 6/ea. D) Schematic of skin biomechanical testing (left). Representative force‐displacement curve of dorsal skin in different groups (middle). Relative quantification of the skin Young's modulus (right). *n* = 6/ea. E) (i) AFM morphology of dermal collagen fibers. (ii) Corresponding AFM property maps. (iii) Quantification of the fibers Young's modulus. *n* = 6/ea. F) Representative WB and quantification of α‐SMA, Postn, Col1A1 and Col3A1 in skin samples of each group. *n* = 6/ea. G) Representative immunofluorescent micrographs of skin stained with indicated antibodies in the corresponding groups. Data are presented as mean ± SEM. B, C, D, E and F by one‐way ANOVA followed by Bonferroni post hoc test. ns = no significance, *p < 0.05, **p < 0.01, ***p < 0.001 compared with corresponding groups.

To investigate the role of Chi3L1 in fibrosis in vivo, we generated Chi3L1 KO mice with a C57BL/6 background and compared the fibrotic changes in wild‐type (WT) and KO mice in phosphate‐buffered saline (PBS) control (CTR) and BLM‐SSc mice, a widely recognized experimental model of SSc (**Figure** **4**A).^[^ ^32^, ^33^ ^]^

First, we examined alterations in Chi3L1 expression in BLM‐SSc mice. Immunofluorescence staining, WB, and qPCR analysis validated Chi3L1 overexpression in the skin of BLM‐SSc mice (Figure S6A–C, Supporting Information). Additionally, ELISA confirmed elevated serum Chi3L1 and IL‐11 levels in BLM‐SSc mice, with IL‐11 levels decreasing upon CHI3L1 deletion (Figure S6D, E, Supporting Information).^[^ ^30^ ^]^

Subsequently, the changes in skin fibrosis were assessed. The results obtained from high‐frequency skin ultrasound demonstrated a significant increase in dermal thickness in BLM‐SSc WT mice compared with that in the PBS group, whereas this change was reversed in BLM‐SSc KO mice (Figure 4B).^[^ ^34^ ^]^ HE and Masson's trichrome staining verified the lack of structural differences between the skin of WT and KO mice in the PBS group. In contrast, BLM‐SSc KO mice exhibited a noticeable improvement in dermal fibrosis with reduced dermal thickness compared with that in BLM‐SSc WT mice (Figure 4C). Skin hydroxyproline content was also significantly lower in BLM‐SSc KO mice (Figure 4C). Moreover, we conducted biomechanical testing on the dorsal skin of the mice to quantify skin elasticity.^[^ ^35^ ^]^ The force‐displacement curve showed that BLM‐SSc KO mouse skin required less stress to rupture and withstood longer stretching distances, indicating greater elasticity with a lower Young's modulus than that in BLM‐SSc WT mouse skin (Figure 4D). To further assess the relationship between fibrillar pattern and skin mechanics, AFM was employed to examine the fibrillar bundles and Young's modulus of mouse skin.^[^ ^36^ ^]^ Compared with that in the PBS group, BLM‐SSc WT mice exhibited a more disorganized fiber ultrastructure with a higher Young's modulus, whereas these conditions were distinctly ameliorated in BLM‐SSc KO mice (Figure 4E). This result proved that Chi3L1 KO ameliorated fibrosis in BLM‐SSc mice at a more microscopic level. qPCR analysis demonstrated that, compared with that in BLM‐SSc WT mice, the expression of fibrosis‐related genes (*α‐SMA*, *Postn*, *Col1A1*, *Col1A3*, *Fn1*, *Timp1*, *Vimentin*, and *connective tissue growth factor*, *Ctgf*) were downregulated in BLM‐SSc KO mice. In contrast, collagen degradation‐related genes (*matrix metalloproteinase 3*, *Mmp3*, and *Mmp9*) were upregulated (Figure S7A, Supporting Information).^[^ ^37^ ^]^ WB analysis and immunofluorescence images further verified the remarkable downregulation of α‐SMA, Postn, Col1A1, and Col3A1 in BLM‐SSc KO mice (Figure 4F,G; Figure S7B, Supporting Information).

From another perspective, Chi3L1 deficiency also obviously reduced the extent of fibrosis and hydroxyproline content in the lungs of BLM‐SSC mice (Figure S8A,B, Supporting Information).^[^ ^33^ ^]^ ELISA and qPCR analyses revealed a decline in Col1A1 expression in the lungs of BLM‐SSc KO mice compared with BLM‐SSc WT mice, suggesting lung fibrosis mitigation (Figure S8C,D, Supporting Information). Collectively, these data suggest that Chi3L1 promotes fibrosis in vivo.

### 2.5 Chi3L1 Activates SSc DFs Via Interacting with IL‐17RA to Further Initiate NF‐kB and MAPK Pathways

**Figure 5:**
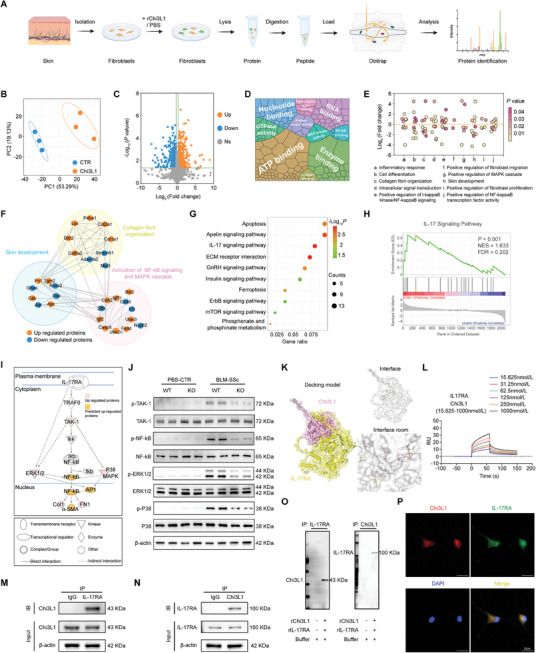
Chi3L1 activates SSc DFs via interacting with IL‐17RA to further initiate NF‐kB and MAPK pathways. A) Schematic overview of proteomics. B) PLS‐DA of proteomic profiling between Chi3L1 and control group. C) Volcano plots of of DEPs. D) Enriched GO terms by MF. Area represents enrichments. E) Primary BP in the DEPs. The clusters are referred to BP terms. Each dot represents a single DEP. F) Cytoscape analysis of protein‐protein interactions network. G) Signaling pathway classification according to KEGG terms. **H**) GSEA analysis via Reactome database. I) IPA of the DEPs. J) Representative WB images of indicated proteins in corresponding groups. K) Surface diagram of the docking model and their interfacing residues between Chi3L1 and IL‐17RA protein (Human source). L) The direct interaction between Chi3L1 and IL‐17RA determined by SPR. M, N) Cellular CO‐IP assays of Chi3L1 and IL‐17RA. SSc DFs lysates were immunoprecipitated with antibody against IL‐17RA, immunoblot with Chi3L1 antibody (M) and vice versa (N). O) CO‐IP analysis of rChi3L1 and rIL‐17RA in a cell‐free system. P) Immunofluorescence co‐localization of Chi3L1 and IL‐17RA in SSc DFs.

To further explore the molecular mechanisms by which Chi3L1 promotes fibrosis in SSc, we conducted a proteomic analysis of DFs with or without rChi3L1 stimulation (**Figure** **5**A). Partial least squares discrimination analysis (PLS‐DA) revealed distinct protein profiling between the Chi3L1 and control groups (Figure 5B). The Chi3L1 group exhibited significant upregulation of 470 proteins and downregulation of 379 proteins relative to the CTR group (Figure 5C). To gain deeper insights into the differentially expressed proteins (DEPs), we performed GO analysis. The GO terms illustrated that the DEPs exerted protein binding and kinase activities, then participated in BP with regard to NF‐kB and MAPK signaling pathway regulation, fibroblast proliferation, differentiation, migration, and collagen synthesis (Figure 5D,E; Figure S9A, Supporting Information). Simultaneously, protein–protein interaction network analysis confirmed that proteins involved in activating NF‐kB and MAPK pathways were highlighted in the cooperation network among DEPs, which is closely linked to collagen synthesis and skin development (Figure 5F). These pieces of evidence suggested that Chi3L1 could promote SSc fibrosis.

Subsequently, pathway analysis was performed to pinpoint the major pathways affected in DFs treated with rChi3L1. KEGG analysis showed that the apelin and IL‐17 signaling pathways were highly enriched in the Chi3L1 group (Figure 5G). GSEA also revealed that the IL‐17 signaling pathway was significantly activated in the Chi3L1 group (Figure 5H). Extensive studies have affirmed that IL‐17 signaling triggers transcription factors to induce gene expression through the NF‐kB and MAPK pathways, which regulate various cellular activities, including apoptosis, proliferation, and differentiation.^[^ ^38^, ^39^, ^40^ ^]^ Aberrations in the NF‐kB and MAPK pathways are widely involved in the pathogenesis of inflammatory, fibrotic, and neoplastic diseases.^[^ ^41^, ^42^ ^]^ Furthermore, ingenuity pathway analysis (IPA) indicated that Chi3L1could bind to IL‐17RA, the ubiquitously expressed primary receptor of the IL‐17 pathway, to further activate the downstream NF‐kB and MAPK pathways (Figure 5I).^[^ ^43^ ^]^ Within the IL‐17 pathway, TGF‐β‐activated kinase 1 (TAK‐1) activated by polyubiquitinated TNF receptor‐associated factor 6 (TRAF6), not only leads to NF‐kB activation but also contributes to P38/ERK MAPK pathway activation. Our WB results also showed that the enhanced phosphorylated levels of TAK‐1, NF‐kB, ERK1/2, and P38 were diminished after Chi3L1 KO under BLM‐SSc conditions (Figure 1J; Figure S9B, Supporting Information). Meanwhile, GO analysis of the DEGs in the fibroblasts in our scRNA‐seq also suggested similar BP terms including the regulation of NF‐kB and P38/ERK MAPK pathways. The corresponding proteins related to these pathways, including IL‐17RA, were also upregulated in patients with SSc, consistent with previous studies (Figure S9C, D, Supporting Information).^[^ ^44^ ^]^ The above evidence suggests that NF‐kB and P38/ERK MAPK pathway activation may be the major pathway involved in Chi3L1‐mediated fibrosis in SSc.

Next, to verify that Chi3L1 might interact with IL‐17RA to further activate NF‐kB and MAPK pathways, we conducted rigid protein–protein docking between Chi3L1 and IL‐17RA.^[^ ^45^ ^]^ Notably, hydrogen bonds were formed between Chi3L1 and IL‐17RA at specific amino acid residue sites like HIS 149‐GLU 241 and ASP 199‐SER 329, indicating the development of a stable docking model. (Figure 5K; Table S2, Supporting Information). Furthermore, Surface plasmon resonance (SPR) revealed the direct interaction between Chi3L1 (15.625–1000 nmol L^−1^) and IL‐17RA (Figure 5L; Table S3, Supporting Information).^[^ ^46^ ^]^ Co‐immunoprecipitation (Co‐IP) assays suggested that Chi3L1 interacts with IL‐17RA in cell lysates obtained from SSc DFs treated with Chi3L1 (Figure 5M,N). The co‐incubation of rChi3L1 and recombinant IL‐17RA in a cell‐free system also confirmed that Chi3L1 interacted with IL‐17RA (Figure 5O). Additionally, immunocytochemistry showed the co‐localization of Chi3L1 and IL‐17RA in SSc DFs treated with Chi3L1 (Figure 5P). Coincidentally, both docking models and Co‐IP proved that Chi3L1 can indeed bind to IL‐17RA in mice as well (Figure S10 and Table S4, Supporting Information).

In summary, our findings indicated that Chi3L1 could directly interact with IL‐17RA, resulting in the activation of the NF‐kB and MAPK pathways.

### 2.6 Chi3L1 Activates SSc DFs Through IL‐17RA‐Dependent NF‐kB and MAPK Pathways

**Figure 6:**
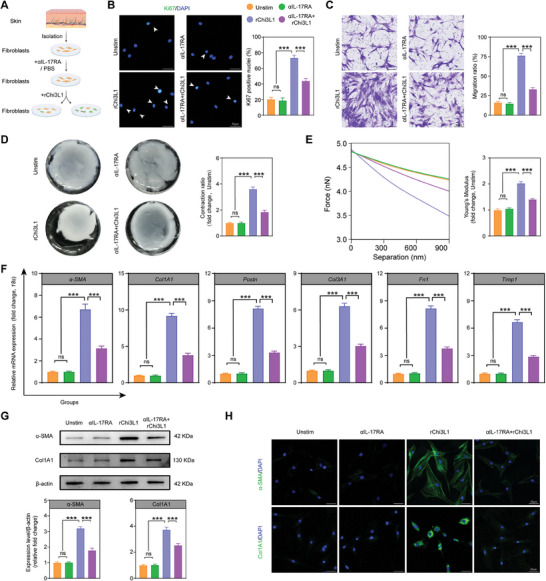
Chi3L1 activates SSc DFs through IL‐17RA‐dependent NF‐kB and MAPK pathways. A) Schematic of the experimental protocol. B) Representative immunofluorescent staining images with Ki67 at 24 h after rChi3L1 treatment. Quantification is shown on the right. *n* = 6/ea. C) Representative images and quantification of transwell assay at 24 h with rChi3L1 stimulation. *n* = 6/ea. D) Representative images and relative quantification of collagen gel contractility assay after 24 h of rChi3L1 administration. *n* = 6/ea. E) Representative force‐separation curve of DFs after stimulation. Relative quantification of the cell Young's modulus is on the right. *n* = 6/ea. F) Relative fold change of indicated mRNA expression level in corresponding groups. *n* = 6/ea. G) Representative WB results and relative quantification of α‐SMA and Col1A1. *n* = 6/ea. H) Representative immunofluorescence micrographs of DFs stained with indicated antibodies after administration. Data are presented as mean ± SEM. B, C, D, E, F and G, by one‐way ANOVA followed by Bonferroni post hoc test. ns = no significance, *p < 0.05, ***p < 0.001 compared with corresponding groups.

Next, we investigated whether Chi3L1‐induced SSc DFs activation was primarily mediated by initiating the NF‐kB and MAPK pathways, dependent on IL‐17RA, in vitro. Brodalumab, a human monoclonal immunoglobulin G antibody approved for treating psoriasis vulgaris, psoriatic arthritis, pustular psoriasis, and psoriatic erythroderma, has a high affinity for IL‐17RA.^[^ ^47^ ^]^ SSc DFs were pretreated with fully human anti‐IL‐17RA monoclonal antibody (anti‐IL‐17RA mAb) or PBS, followed by rChi3L1 treatment (**Figure** **6**A). Administration of anti‐IL‐17RA mAb significantly inhibited the key phosphorylated proteins of the NF‐kB and MAPK pathways in SSc DFs that were upregulated by rChi3L1 (Figure S11A, Supporting Information). Additionally, immunofluorescence images indicated the suppression of NF‐kB nuclear translocation (Figure S11B, Supporting Information). Treatment with anti‐IL‐17RA mAb also abolished the enhanced proliferation and migration capacity of SSc DFs mediated by Chi3L1 (Figure 6B,C; Figure S12A,B, Supporting Information). Meanwhile, the strengthened capacity of SSc DF collagen gel matrix contraction and stiffness induced by Chi3L1 were attenuated by anti‐IL‐17RA mAb (Figure 6D,E). Furthermore, the upregulated expression of fibrosis‐related genes *α‐SMA*, *Col1A1*, *Postn*, *Col3A1*, *Fn1*, and *Timp1* under Chi3L1 stimulation was decreased by pretreatment with anti‐IL‐17RA mAb (Figure 6F). Consistently, WB and immunofluorescence results demonstrated that anti‐IL‐17RA mAb repressed Chi3L1‐induced myofibroblast differentiation and collagen synthesis in SSc DFs (Figure 6G,H; Figure S12C, Supporting Information). These findings provide compelling support for Chi3L1‐mediated SSc DF activation predominantly occurring through the IL‐17RA‐dependent initiation of the NF‐kB and MAPK pathways.

### 2.7 Anti‐IL‐17RA mAb Ameliorates Fibrosis in BLM‐SSc Mice

**Figure 7:**
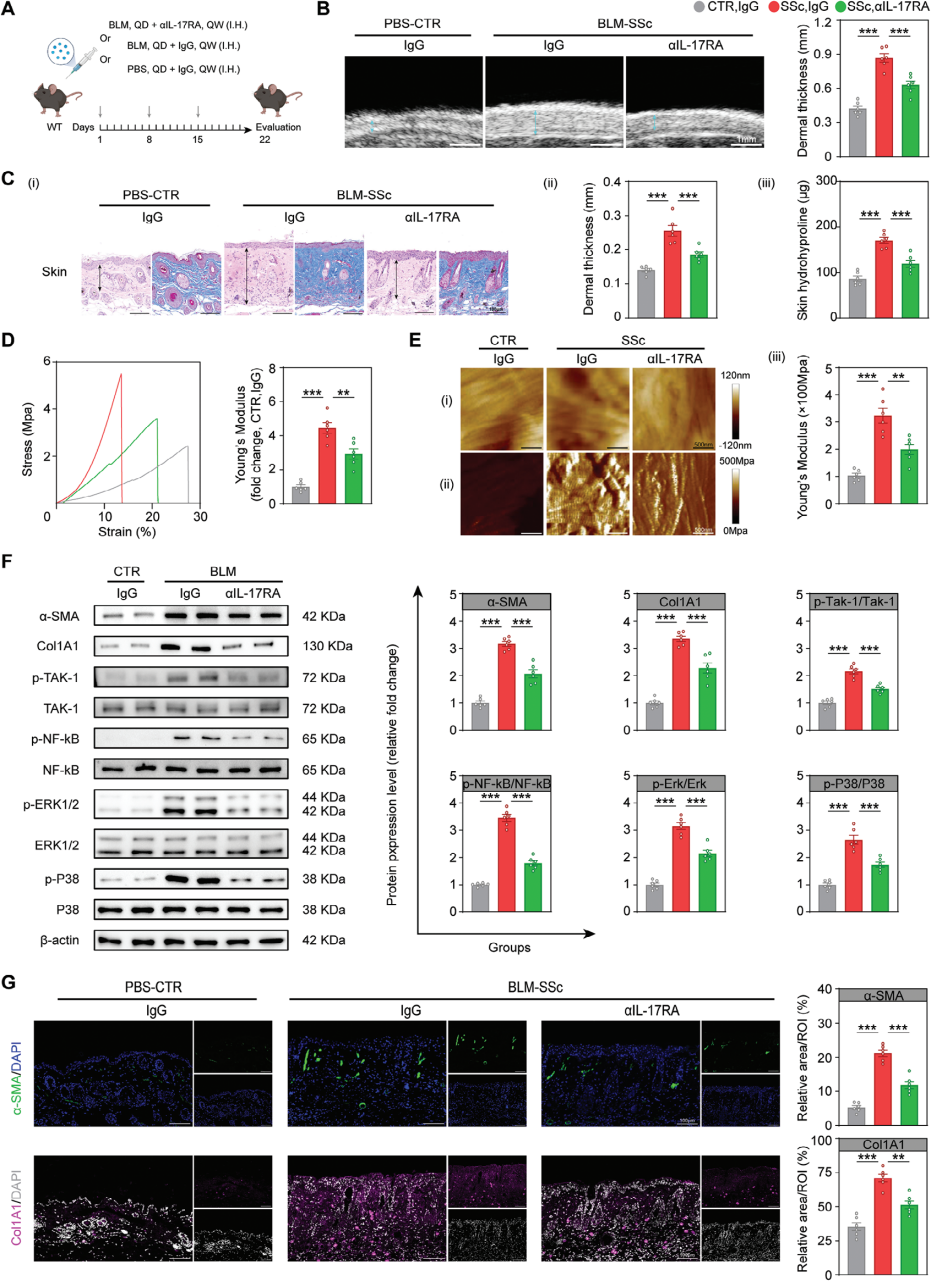
Anti‐IL‐17RA mAb ameliorates fibrosis in BLM‐SSc mice. A) Diagram of the experimental protocol. QW, once a week. B) Representative skin ultrasound images in corresponding groups. Quantification of dermal thickness is shown on the right. *n* = 6/ea. C) (i) Representative HE (left) and Masson (right) staining of skin corresponding groups. (ii) Quantification of dermal thickness. (iii) Hydroxyproline contents of skin samples. *n* = 6/ea. D) Representative force‐displacement curve of dorsal skin in different groups. Relative quantification of the skin Young's modulus is on the right. *n* = 6/ea. E) (i) AFM morphology of dermal collagen fibers. (ii) Corresponding AFM property maps. (iii) Quantification of the fibers Young's modulus. *n* = 6/ea. F) Representative WB images and relative quantification of indicated proteins in skin samples of each group. *n* = 6/ea. G) Representative immunofluorescence images of skin stained with indicated antibodies in corresponding groups. Data are presented as mean ± SEM. B, C, D, E and F by one‐way ANOVA followed by Bonferroni post hoc test. **p < 0.01, ***p < 0.001 compared with corresponding groups.

To further explore the effects of anti‐IL‐17RA mAb on the development of SSc in vivo, we administered anti‐IL‐17RA mAb weekly along with BLM (**Figure** **7**A). Anti‐IL‐17RA mAb reduced dermal thickness and collagen content in the skin of BLM‐SSc mice compared with isotype control IgG (Figure 7B,C). Simultaneously, biomechanical experiments demonstrated that treatment with anti‐IL‐17RA mAb significantly improved dorsal skin elasticity in BLM‐SSc mice (Figure 7D). Furthermore, AFM indicated that anti‐IL‐17RA mAb reversed the deterioration of collagen fiber ultrastructure in BLM‐SSc mice, with a reduction in the increased rigidity (Figure 7E). Anti‐IL‐17RA mAb administration also downregulated fibrosis‐related gene (*α‐SMA*, *Postn*, *Col1A1*, *Col3A1*, *Ctgf*, *Fn1*, *Timp1*, and *Vimentin*) expression in BLM‐SSc mice and upregulated the expression of collagen degradation‐related genes (*Mmp3* and *Mmp9*) (Figure S13, Supporting Information). WB and immunofluorescence analysis further corroborated the decrease in α‐SMA and Col1A1 expression in the skin of BLM‐SSc mice treated with anti‐IL‐17RA mAb (Figure 7F,G). Notably, anti‐IL‐17RA mAb also downregulated the key phosphorylated proteins of the NF‐kB and MAPK pathways in BLM‐SSc mice (Figure 7F). In addition, anti‐IL‐17RA mAb reduced the fibrosis score, hydroxyproline content, and Col1A1 expression in the lungs of BLM‐SSc mice (Figure S14, Supporting Information). In conclusion, anti‐IL‐17RA mAb significantly attenuated BLM‐induced fibrosis by inhibiting NF‐kB and MAPK signaling pathways.

## 3 Discussion

**Figure 8:**
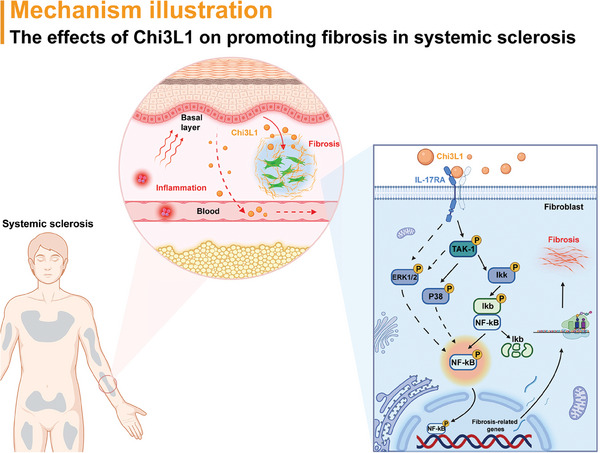
A proposed model of how Chi3L1 promotes fibrosis in SSc. SSc triggers sustainable and complicated inflammation process, possibly inducing the enrichment of basal cells with high expression of Chi3L1. Chi3L1 promotes fibrosis in SSc primarily via activating fibroblasts. Mechanistically, Chi3L1 directly interacts with IL‐17RA on fibroblasts, which in turn activates downstream NF‐kB and MAPK pathways, thereby upregulates fibrosis‐related genes, ultimately leading to enhancement of differentiation into myofibroblasts and detrimental collagen‐rich ECM.

In this study, we identified a cluster of basal cells with high Chi3L1 expression that may play a crucial role in SSc fibroblast activation. Bioinformatics analysis and co‐staining confirmed the enrichment of these keratinocytes in patients with SSc. *Chi3L1^hi^* basal cells exhibited active protein synthesis and secretion processes, and Chi3L1 overproduction enabled fibroblast activation. To the best of our knowledge, this is the first study to elucidate the effects of Chi3L1 in promoting fibrosis in SSc. Mechanistically, Chi3L1 interacts with IL‐17RA, subsequently activating downstream NF‐kB and MAPK pathways, thereby promoting fibroblasts differentiation into myofibroblasts and collagen synthesis (**Figure** **8**). Furthermore, we demonstrated the efficacy of anti‐IL‐17RA mAb in a murine model of SSc, suggesting that Brodalumab treatment is a novel and promising strategy for improving the outcomes of patients with SSc.

Prior research has indicated the significant role of keratinocytes in SSc, as they can activate fibroblasts characterized by increased expression of α‐SMA and Col1A1 in a TGF‐β‐independent manner.^[^ ^8^, ^12^ ^]^ However, the specific mechanisms underlying this process remain unclear. In the present study, we used scRNA‐seq to obtain a comprehensive overview of SSc skin cells. We identified a subgroup of basal cells characterized by high Chi3L1 expression enriched in SSc that exhibited significant interactive potential with inactive fibroblasts. GO terms and GSEA analysis revealed that *Chi3L1^hi^* basal cells were influenced by inflammatory factors such as TNF‐α, suggesting that the complex inflammatory environment in SSc might impact its enrichment preference. *Chi3L1^hi^* basal cells simultaneously displayed positive protein synthesis and secretion.

Chi3L1 is secreted by various structural and immune cells and participates in multiple tissue remodeling and fibrotic diseases.^[^ ^16^ ^]^ For instance, Chi3L1 plays a pro‐fibrotic role in mammalian pulmonary fibrosis by promoting alternative macrophage activation, fibroblast proliferation, and ECM deposition.^[^ ^18^ ^]^ Additionally, elevated serum Chi3L1 levels in hepatitis B virus (HBV) infection serve as a feasible biomarker for advanced liver fibrosis in patients with HBV‐related liver fibrosis.^[^ ^48^ ^]^ Previous studies have shown that patients with SSc have increased serum Chi3L1 levels compared with those in HCs. Moreover, the skin cells of HCs barely secrete Chi3L1; however, in SSc, particularly in its early stages, endogenous Chi3L1 secretion occurs, although the specific source remains unclear.^[^ ^21^, ^49^ ^]^ Based on clues from transcriptome data, our results confirmed that Chi3L1 was predominantly expressed by basal cells among all cells derived from the skin. Chi3L1 was highly expressed in the skin and serum of patients with SSc. Moreover, increased serum Chi3L1 levels in SSc were associated with MRSS and lung function, suggesting its relevance to fibrosis and utility as a potential serum biomarker to reflect the extent of fibrosis in SSc. Furthermore, our findings confirmed that Chi3L1 could enhance the differentiation of SSc DFs into myofibroblasts. Chi3L1 may serve as a bridge between keratinocyte and fibroblast activation. In contrast, Chi3L1 KO mice exhibited fibrosis alleviation in a BLM‐induced SSc model. Subsequently, we employed proteomics to delineate the downstream molecular mechanism of Chi3L1‐induced fibroblast activation and further demonstrated that Chi3L1 could directly bind to IL‐17RA, activating the downstream NF‐kB and MAPK signaling pathways. This leads to the activation of fibroblast and significantly increases collagen synthesis. The roles of the MAPK and NF‐kB pathways in fibrotic diseases have been widely reported.^[^ ^41^, ^42^ ^]^ Among the NF‐kB transcription factor family, not only p65 but also the overexpressed cREL has been shown to regulate distinct transcriptional and functional profiles that drive fibroblast matrix production in SSc.^[^ ^50^, ^51^ ^]^

Brodalumab is an effective IL‐17RA antagonist approved for treatment against severe psoriasis.^[^ ^52^ ^]^ The effectiveness of Brodalumab in SSc has not been definitively established, and our research demonstrates an obvious inhibitory effect of Brodalumab on Chi3L1‐induced fibroblast activation. Therefore, we administered Brodalumab to a BLM‐SSc murine model and observed a remarkable anti‐fibrotic effect. A recent single‐arm, open‐label, phase I trial enrolling eight patients with SSc treated with Brodalumab similarly demonstrated improved clinical SSc features, consistent with our research findings.^[^ ^53^ ^]^

Our study also had limitations. It remains unclear whether the elevated Chi3L1 levels in SSc serum are solely attributable to overproduction by basal cells in the skin. However, we have provided unambiguous evidence illustrating the aberrant expression of Chi3L1 in the basal cells of patients with SSc, as well as the pro‐fibrotic role and mechanism of Chi3L1 in SSc, which holds significant clinical relevance.

Overall, our study delves deep into the role of basal cells in SSc fibrosis and reveals a novel function of Chi3L1 in promoting fibrosis in SSc. Mechanistically, Chi3L1 binds to IL‐17RA on fibroblasts, triggering downstream NF‐kB and MAPK pathways, that ultimately promotes fibroblast activation and excessive collagen‐rich ECM deposition. More broadly, our work suggests that anti‐IL‐17RA mAb has translational potential for improving clinical outcomes in patients with SSc.

## 4 Experimental Section

### Patient Involvement

Seventy‐two participants provided their clinical information and serum samples (Table S1, Supporting Information). Skin biopsies (4 mm) were collected from six participants for scRNA‐seq (Table S5, Supporting Information). The SSc DFs were derived from the forearms of six patients (Table S6, Supporting Information). All patients with SSc met the 2013 American College of Rheumatology/European League against Rheumatism classification criteria.^[^ ^2^ ^]^ All study participants provided informed consent for their inclusion in the study. This research received approval from the Ethics Committee of Zhongshan Hospital, Fudan University.

### scRNA‐seq and Analysis

Dorsal forearm skin biopsies were obtained from the study participants (Table S5, Supporting Information). Tissues were rinsed with Hanks balanced salt solution and digested in 2 mL GEXSCOPE Tissue Dissociation Solution (Singleron Biotechnologies).^[^ ^54^ ^]^ Briefly, the specimens were digested at 37 °C for 15 min and passed through a 40 µm sterile strainer (Corning Life Sciences). The cells were centrifuged at 300 × *g* for 5 min, and the cell pellets were resuspended in 1 mL PBS (HyClone). The cell suspensions were assessed for viability and adjusted to a concentration of 1 × 10^5^ cells mL^−1^. Subsequently, single‐cell suspensions were loaded onto a microfluidic chip (GEXSCOPE Single Cell RNA‐seq Kit; Singleron Biotechnologies), and scRNA‐seq libraries were prepared following the manufacturer's guidelines. Libraries were sequenced using an Illumina NovaSeq 6000 platform (Illumina) with 150‐bp paired‐end reads.

Raw reads were analyzed to produce gene expression profiles with CeleScope v1.5.2 (Singleron Biotechnologies) using default parameters. Quality control, dimensionality reduction, and clustering were carried out using Seurat v3.1.2 according to a previous study.^[^ ^55^ ^]^ Cell clusters were visualized using UMAP with Seurat functions (RunUMAP). The enrichment preferences of the cell clusters were evaluated using Ro/e values.^[^ ^23^ ^]^

DEGs were identified using the FindMarker function with the Wilcox likelihood ratio test. Criteria included expression in more than 10% of cells within a cluster and an average log fold change greater than 0.25. The cell types for each cluster were assigned based on the expression of these DEGs. Heatmaps were used to visualize the typical differential genes within cell clusters.

For pseudo‐temporal trajectory analysis, Monocle2 was employed, utilizing the DDRTree method for dimensionality reduction to map fibroblast subtype differentiation. To analyze the order of gene expression and function during cell state transitions, switch gene analysis was conducted with GeneSwitches (V0.1.0) in R version 3.6.3 (R Foundation for Statistical Computing).

GO, KEGG, and GSEA analyses were employed to forecast the biological functions, cellular components, and potential pathways related to the DEGs, with an adjusted *P*‐value of less than 0.05 regarded as statistically significant. Cell–cell interactions were predicted based on known ligand–receptor pairs using CellphoneDB (v2.1.0; Teichmann Lab).^[^ ^56^ ^]^

### qPCR

Total RNA was extracted from tissues or cells using a Total RNA Isolation Kit (Vazyme) following the manufacturer's protocol. cDNA was prepared using a HiScript III 1st Strand cDNA Synthesis Kit (Vazyme) according to the manufacturer's protocol. qPCR was conducted using Universal SYBR Green Master Mix (Vazyme). The primers used for qPCR are listed in Table S7 (Supporting Information). *18s* was used as the housekeeping gene, and gene expression was calculated using the standard comparative CT method.

### Immunofluorescence Staining

Skin tissues were embedded in optimal cutting temperature compound and sliced into 7 µm sections. The cryosections were then subjected to blocking with 10% goat serum albumin and 0.03% Triton X‐100 for 1 h at room temperature following fixation/permeabilization. Next, The slides were incubated overnight at 4 °C with the specified primary antibodies (Table S8, Supporting Information). Following PBS washes, the sections were incubated with fluorescent secondary antibodies for 2 h at room temperature. Nuclei were counterstained with DAPI (Invitrogen) for 10 min. Fluorescence microscopy was used to view the slides, and fluorescence intensity was analyzed using ImageJ software (1.52 V; National Institutes of Health).

### Histology

Formalin‐fixed and paraffin‐embedded skin and lung tissues were stained with HE. Dermal thickness was measured as the distance between the epidermal–dermal and the dermal–adipose junctions. The extent of lung fibrosis was evaluated semi‐quantitatively based on previously outlined criteria.^[^ ^57^ ^]^ Alternatively, the tissues were stained with Masson's trichrome to assess collagen deposition. All sections were analyzed in a blinded manner.

### WB

Total protein was isolated from cells or skin tissues using RIPA buffer supplemented with a complete protease inhibitor and phosphatase inhibitor (Roche). Protein concentrations were assessed with a Pierce BCA Protein Assay Kit (Thermo Fisher Scientific). Proteins were then separated by SDS‐PAGE and transferred to polyvinylidene difluoride membranes. The membranes were blocked with 5% bovine serum albumin (BSA). Thereafter, the membranes were incubated with the indicated primary antibodies at 4 °C overnight (Table S8, Supporting Information). Subsequently, the membranes were incubated with horseradish peroxidase‐conjugated secondary antibodies for 2 h. The membranes were analyzed using a Tanon instrument (Tanon Science & Technology) and quantified using ImageJ software (1.52 V; NIH).

### ELISA

Chi3L1, IL‐11, and collagen type I levels were measured using specific ELISA kits (Puwang Biotechnology; R&D Systems; Novus Biological, respectively). For the preparation of tissue samples, tissues were exposed to ice‐cold lysis buffer (10 mM pH = 8.0 Tris; 130 mM NaCl 1% TritonX‐100; protease inhibitor cocktail) for 60 min. The samples were then centrifuged, and the supernatant was collected. The experiments were performed according to the manufacturer's guidelines. The absorbance of the plate was measured at 450 nm.

### Fibroblast Isolation

Skin tissues were obtained from the forearms of six patients with SSc (Table S6, Supporting Information) and the dorsum of BLM‐SSc mice. Subsequently, the samples were digested with 0.1% type I collagenase (Worthington Biochemical Corporation) and 0.25% trypsin (Thermo Fisher Scientific) for 1 h at 37 °C with shaking. Following digestion, the supernatants were filtered using a 70 µm nylon mesh and plated for 2 h to allow DF adherence. Afterward, the medium was removed and substituted with fresh culture medium.

### Cell Culture, Stimulation, and Immunostaining

DFs were cultured in DMEM‐F12 supplemented with 10% fetal bovine serum and 1% penicillin/streptomycin at 37 °C in an incubator containing 5% CO_2_ at low passage (<p5). For stimulation, DFs were serum‐starved for 24 h and treated with specific concentrations of rChi3L1 (MedChemExpress) or Brodalumab (MedChemExpress) for an appropriate time.

After treatment, DFs were fixed with 4% paraformaldehyde, permeabilized with 0.03% TritonX‐100 for 20 min, and blocked with 5% BSA in 1% donkey serum for 30 min. DFs were then incubated overnight at 4 °C with the indicated primary antibodies (Table S8, Supporting Information). Next, DFs were stained with a fluorescent secondary antibody for 1 h at room temperature, and nuclei were labeled with DAPI (Invitrogen). Fluorescence microscopy was used to obtain images, and fluorescence intensity was measured using ImageJ software (1.52 V; NIH).

### CCK‐8 Assay

SSc DFs were seeded in 96‐well plates, and cell viability was analyzed using a CCK‐8 kit (Dojindo Laboratories) at the appropriate time intervals. Absorbance was measured at 450 nm.

### LDH Assay

To evaluate cell injury, an LDH release analysis was conducted, as apoptotic or dead cells release stable amounts of LDH. After rChi3L1 treatment, SSc DFs were lysed and assessed using the LDH Cytotoxicity Assay Kit (Yeasen Biotechnology), following the manufacturer's instructions.

### Scratch Assay

SSc DFs reached 100% confluence and serum‐starved overnight. The scratch wounds were generated using a sterile p1000 pipette tip (Gilson). To prevent cell proliferation, DFs were pretreated with 10 µg mL^−1^ mitomycin C (Sigma–Aldrich). After scratching, the DFs were stimulated or left untreated for 24 h. Wound closure was expressed as the percentage of wound reduction relative to the original wound. The wound area was quantified using ImageJ software (1.52 V; NIH).

### Transwell Assay

Migratory assays were performed using an 8 µm Transwell system (Corning Costar; Corning Life Sciences). DFs were seeded at 3 × 10^4^ cells/well in serum‐free medium in the top chamber, with complete medium placed in the bottom wells. After 24 h of incubation, non‐migratory cells remaining in the top chamber were removed. The migratory DFs were then fixed with 4% paraformaldehyde, and stained with 0.01% Crystal Violet (Sigma–Aldrich). The migration was quantified by examining six random fields under a microscope.

### Collagen Gel‐Based Contractility Assay

The contractility of Chi3L1‐mediated SSc DFs was evaluated using a Collagen Gel‐based 48‐well Cell Contraction Assay Kit (Cell Biolabs) following the manufacturer's protocol.^[^ ^58^ ^]^ Images were captured using a digital camera at 0 and 24 h. The size of the gels was measured and normalized to their respective well sizes using ImageJ software (1.52 V; NIH).

### AFM

AFM (FastScan Bio; Bruker) with an MLCT‐O10 probe (Bruker) was used to investigate the mechanical properties of the cells at a scanning rate of 1.0 Hz and peak‐force amplitude of 100 nm. NanoScope Analysis software (version 1.8; Bruker) was used to analyze the obtained force‐distance curves. Three measurements were conducted on each cell to determine Young's modulus. Measurements performed around the nucleus were less affected by artifacts owing to substrate stiffness.^[^ ^27^, ^59^ ^]^ Regions distal to the nucleus were avoided. The force‐distance curves were corrected, and the Hertzian (Spherical) model was fitted to extract the apparent Young's modulus. The Poisson's ratio of the samples was 0.3.^[^ ^36^ ^]^

An AC240 probe (Olympus Corporation) was used to determine tissue surface morphology. The scanning rate was set to 2.0 Hz, the peak‐force setpoint was 30 nN, and the peak‐force amplitude was 70 nm. The deflection sensitivity was calibrated using a sapphire model. NanoScope Analysis software (version 1.8; Bruker) was used to analyze the data. Each scan generated a morphological image at a resolution of 256 × 256 pixels.

### Mice, BLM‐SSc Model, and Drug Administration

WT C57BL/6 mice (female, 6 weeks) were purchased from JieSiJie Laboratory Animal Ltd. (Shanghai, China). The CRISPR/Cas9 technique was used to generate Chi3L1 KO mice on a C57BL/6 background that were maintained as previously described.^[^ ^32^ ^]^ Mice were bred under specific pathogen‐free conditions. All animal experiments were conducted in strict accordance with the Animal Care and Use Committee of Shanghai Jiao Tong University. All procedures complied with the National Institutes of Health Guidelines for the Care and Use of Laboratory Animals.

To create BLM‐SSc mice, BLM (Nippon Kayaku) was dissolved in PBS to a concentration of 0.5 mg mL^−1^ and injected subcutaneously into the shaved backs of mice at a dose of 100 µL for 21 successive days. Mice receiving PBS injections served as controls for the BLM‐SSc group.

To assess the impact of blocking IL‐17RA, the anti‐IL‐17RA monoclonal antibody Brodalumab (MedChemExpress) or mouse IgG1 kappa isotype control (eBioscience) was subcutaneously injected at a dose of 200 µg every 7th day (days 1, 8, and 15), with daily administration of BLM for 3 weeks (day 1 to day 21). The mice were euthanized through carbon dioxide inhalation, and cervical dislocation was carried out afterward.

### Skin Ultrasound

High‐frequency skin ultrasound was conducted using a Vevo 2100 instrument (FUJIFILM VisualSonics) to assess dermal thickness in vivo.^[^ ^34^ ^]^ Full‐thickness dorsal skin images were obtained from the right side of the mouse spine and dermal thickness was measured in a blinded manner.

### Hydroxyproline Assay

The collagen content in the skin and lung tissues of mice was measured using a QuickZyme Total Collagen Assay (QuickZyme Biosciences), following the manufacturer's guidelines. Total left lung and skin samples were used.

### Mechanical Assessment

A tissue mechanics testing machine (Mach‐1; Biomomentum) was used to investigate the mechanical properties of the skin.^[^ ^35^ ^]^ The shaved dorsal skin of the mice was harvested on day 22 and prepared in a dog bone shape. A high‐precision ruler was used to measure the skin length and cross‐sectional area. Thereafter, the skin was fixed, a pre‐load of 0.01 N was applied, and each skin sample was cyclically elongated using loads from 0.02 to 0.04 N at a speed of 0.02 mm s^−1^ for 10 cycles. Subsequently, the skin samples were subjected to failure tests at an elongation rate of 0.02 mm s^−1^. The load‐elongation behavior and failure mode of the skin samples were recorded. Mach‐1 Analysis software (Biomomentum) was used to analyze the force‐displacement curves. The structural characteristics of the skin samples were then quantified.

### Proteomics and Analysis

Label‐free proteomics and data‐independent analysis were conducted as previously described.^[^ ^60^ ^]^ Raw mass spectrometry data were processed using Skyline and matched the UniProt database.^[^ ^61^ ^]^ The DEPs with a fold change greater than 1.5 and a *P*‐value below 0.05 were further analyzed using GO terms (https://david.ncifcrf.gov/) and KEGG (https://www.kegg.jp/). GSEA was performed using GSEA software (http://www.gsea‐msigdb.org/). Kinase substrate enrichment analysis was performed using Cytoscape software (https://cytoscape.org/).

### Molecular Docking Analysis

Rigid protein–protein docking of Chi3L1 and IL‐17RA from human/mouse sources was conducted using GRAMM‐X (http://gramm.compbio.ku.edu/). Structural information was acquired from the Protein Data Bank (http://www.rcsb.org/). Pymol (Version 2.4; Schrödinger, Inc) and PDBePISA (https://www.ebi.ac.uk/pdbe/pisa/) were utilized for protein–protein interaction analysis and visualization.^[^ ^45^ ^]^

### Co‐IP

DFs from patients with SSc or BLM‐SSc mice were treated with human or mouse rChi3L1 (MedChemExpress) for 10 min. Next, DFs were gently rinsed with PBS and lysed using immunoprecipitation lysis buffer (2.5 mM pH 7.4 Tris; 150 mM NaCl, 1 mM EDTA, 1% NP40, 5% glycerol, protease inhibitor cocktail). Co‐IP was conducted using a Pierce Co‐IP kit (Thermo Fisher Scientific) following the manufacturer's instructions. For cell‐free in‐tube assays, 5 µg human rChi3L1 (MedChemExpress) and 5 µg human rIL‐17RA (MedChemExpress) were mixed in lysis buffer and shaken at 4 °C overnight before performing Co‐IP.

### SPR Analysis

The direct interaction between Chi3L1 and IL‐17RA was analyzed using a Biacore 8 K system (Cytiva), following the manufacturer's instructions. The IL‐17RA recombinant protein was immobilized on a Series S Sensor Chip CM5 (GE Healthcare Life). Different Chi3L1 concentrations, diluted in running buffer, were injected into the system as the analytes. Interaction parameters were subsequently determined using Biacore evaluation software (Version 2.0; Cytiva).^[^ ^46^ ^]^

### Statistical Analysis

All quantitative data were shown as the mean ± standard error of the mean (SEM). Data normality was examined using the Shapiro–Wilk normality test. Normally distributed data were analyzed using an unpaired Student's *t*‐test or ANOVA followed by Bonferroni post hoc analysis for comparisons between two or multiple groups (>2 groups), respectively. Mann–Whitney non‐parametric tests were used for data with non‐normal distributions. Correlations were assessed using Spearman's correlation. The chi‐squared test was used for categorical data. Statistical significance was considered at *p* < 0.05. Statistical analyses were performed using GraphPad Prism 9.0 (GraphPad Prism Software, Inc., San Diego, CA, USA).

### Ethics Statement

All patients provided written informed consent prior to enrollment in the study, and the study was approved by the Ethics Committee of Zhongshan Hospital, Fudan University (NO. Y2021‐073). Animal studies were strictly performed in compliance with the Animal Care and Use Committee of Shanghai Jiao Tong University (NO. O_A2023071).

## Conflict of Interest

The authors declare no conflict of interest.

## Supporting information



Supporting Information

## Data Availability

The data that support the findings of this study are available from the corresponding author upon reasonable request.
