# Supplementary material for: Aberrant Chitinase 3‐Like 1 Expression in Basal Cells Contributes to Systemic Sclerosis Fibrosis
Source: Adv Sci (Weinh). 2024 Dec 17;12(6):2310169. doi: 10.1002/advs.202310169 (PMC11809421; doi:10.1002/advs.202310169)
Supplement: Supplementary file 1 — Supporting Information [file ADVS-12-2310169-s001.docx]

**Supporting Information**

**Aberrant Chitinase 3-Like 1 Expression in Basal Cells Contributes to Systemic Sclerosis Fibrosis**

Wang *et al*.

This document includes:

Supplemental Figure 1-14

Supplemental Table 1-8

**Supplemental Figures**

**
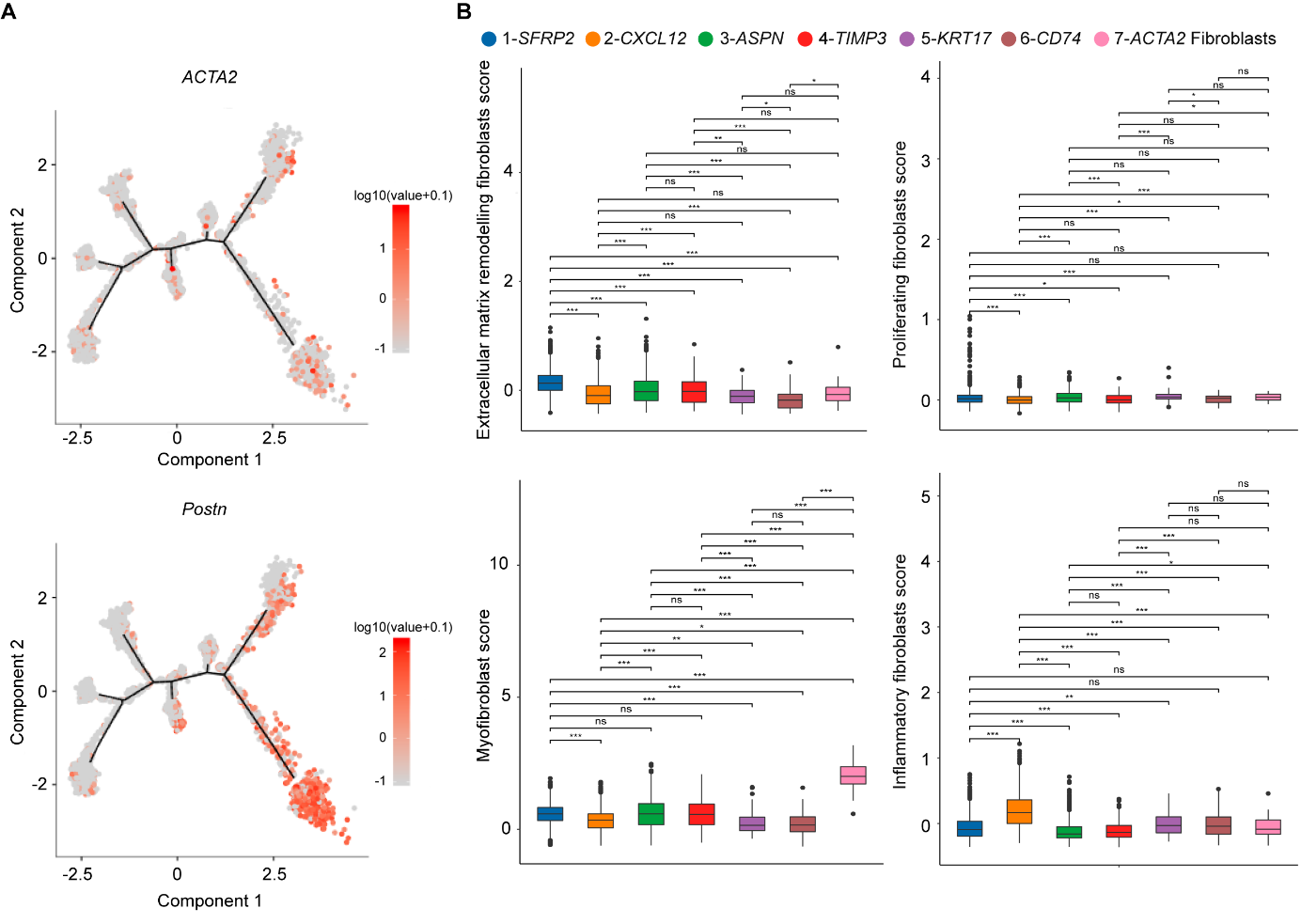
**

**Figure S1. A.** The expression dynamics of selected marker genes indicated by pseudotime analysis. **B.** Fibroblast characteristics scoring. Box plots represented the 25th and 75th percentiles, lines inside the boxes represented the median. Significance was evaluated by two-tailed Mann–Whitney U test, ns=no significance, **P*<0.05, ***P*<0.01, ****P*<0.001 compared with corresponding clusters.

**
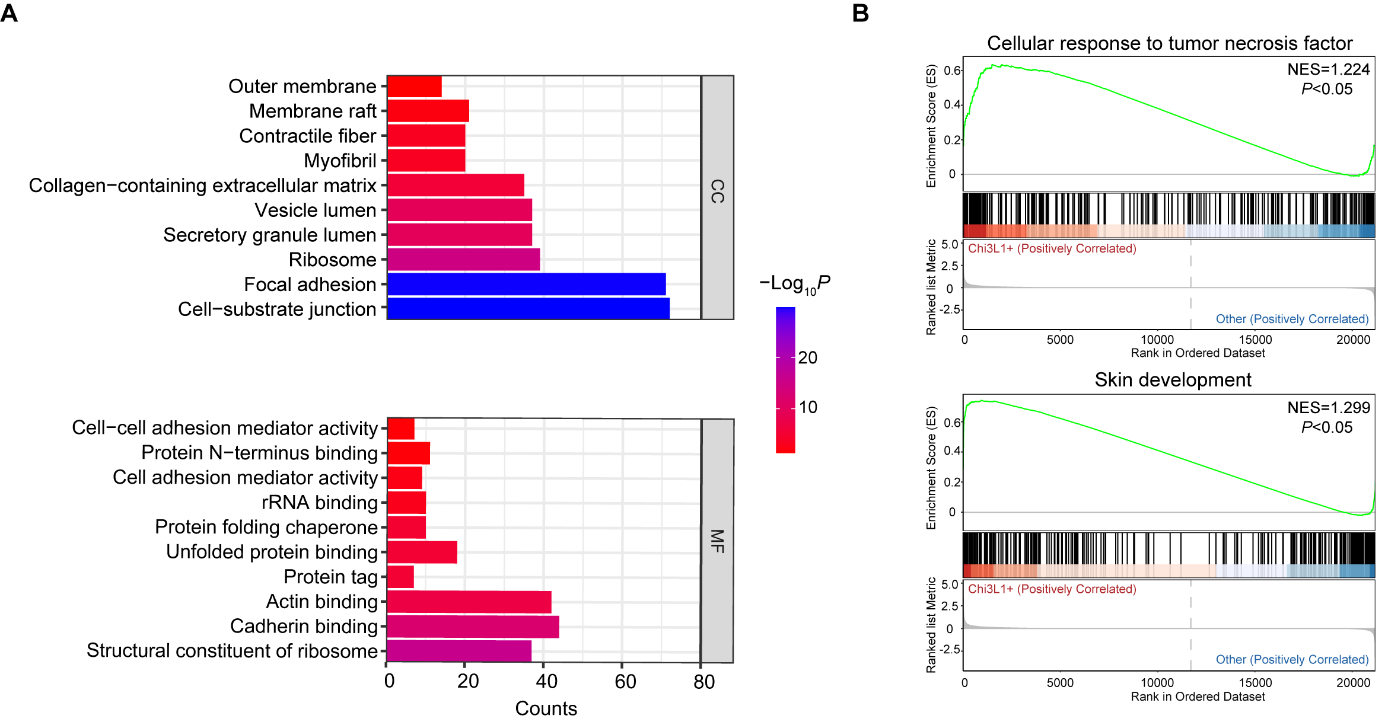
**

**Figure S2. A.** CC and MF analysis of DEGs. **B.** GSEA analysis via GO and KEGG database.

**
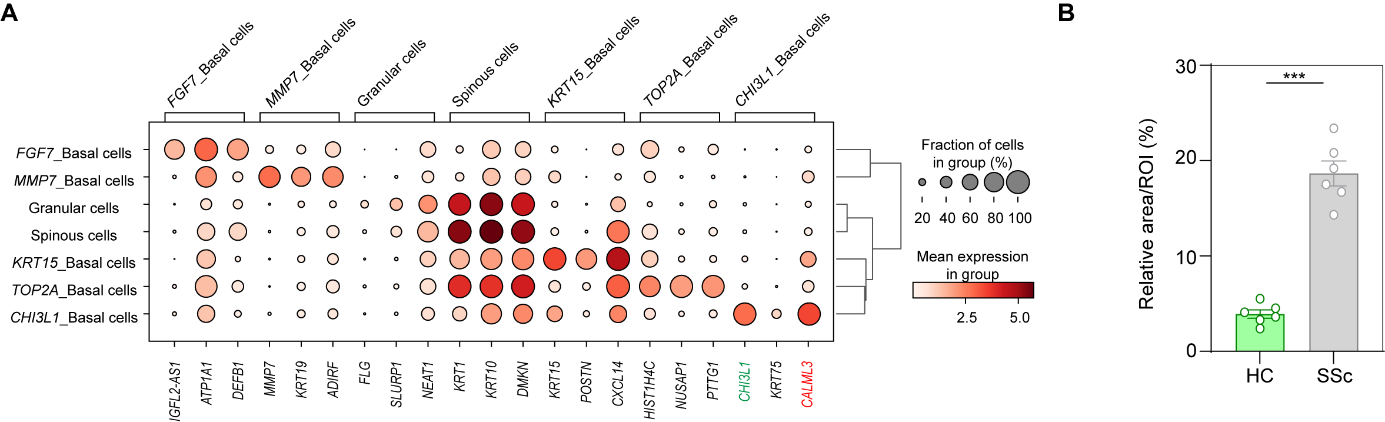
**

**Figure S3. A.** Heatmap of marker genes for keratinocytes. **B.** Relative quantification of immunofluorescence staining. n=6/ea. Data are presented as mean ± SEM. Significance was evaluated by unpaired Student’s *t*-test. ****P* < 0.001.


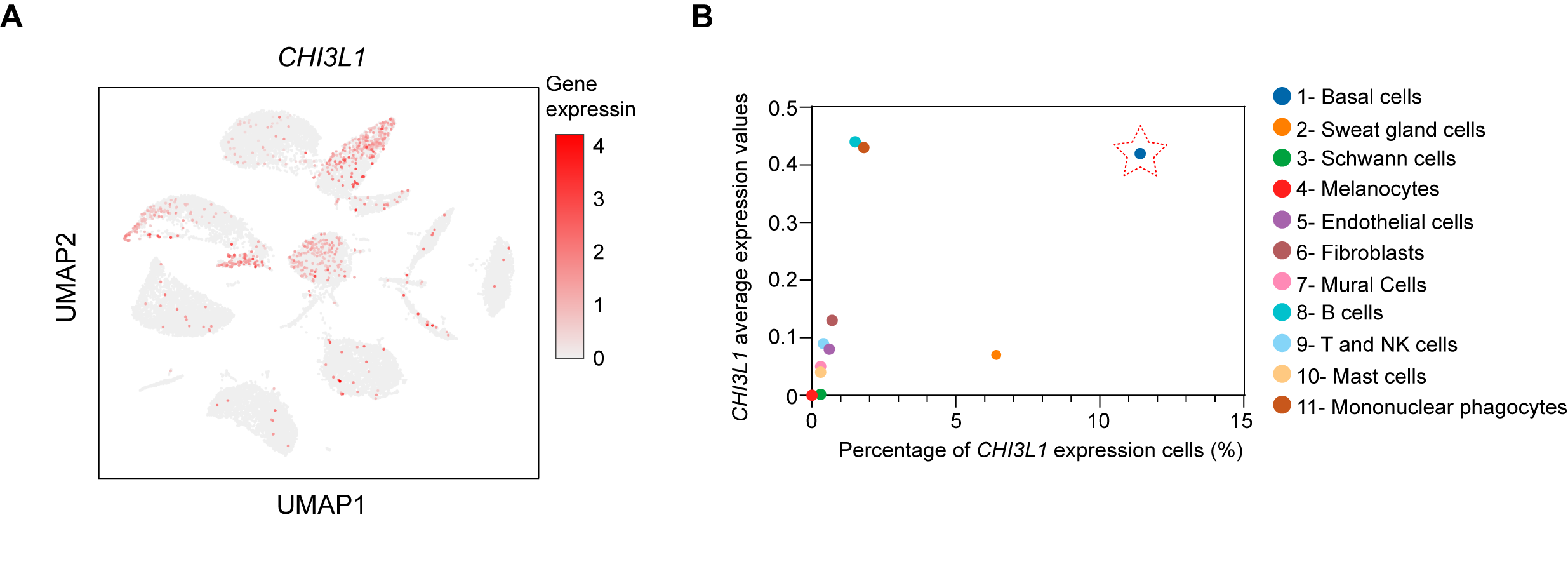


**Figure S4. A.** The UMAP of *Chi3L1* expression. **B.** The average expression levels of *Chi3L1* and the proportion of *Chi3L1* expressing cells across different cell types.


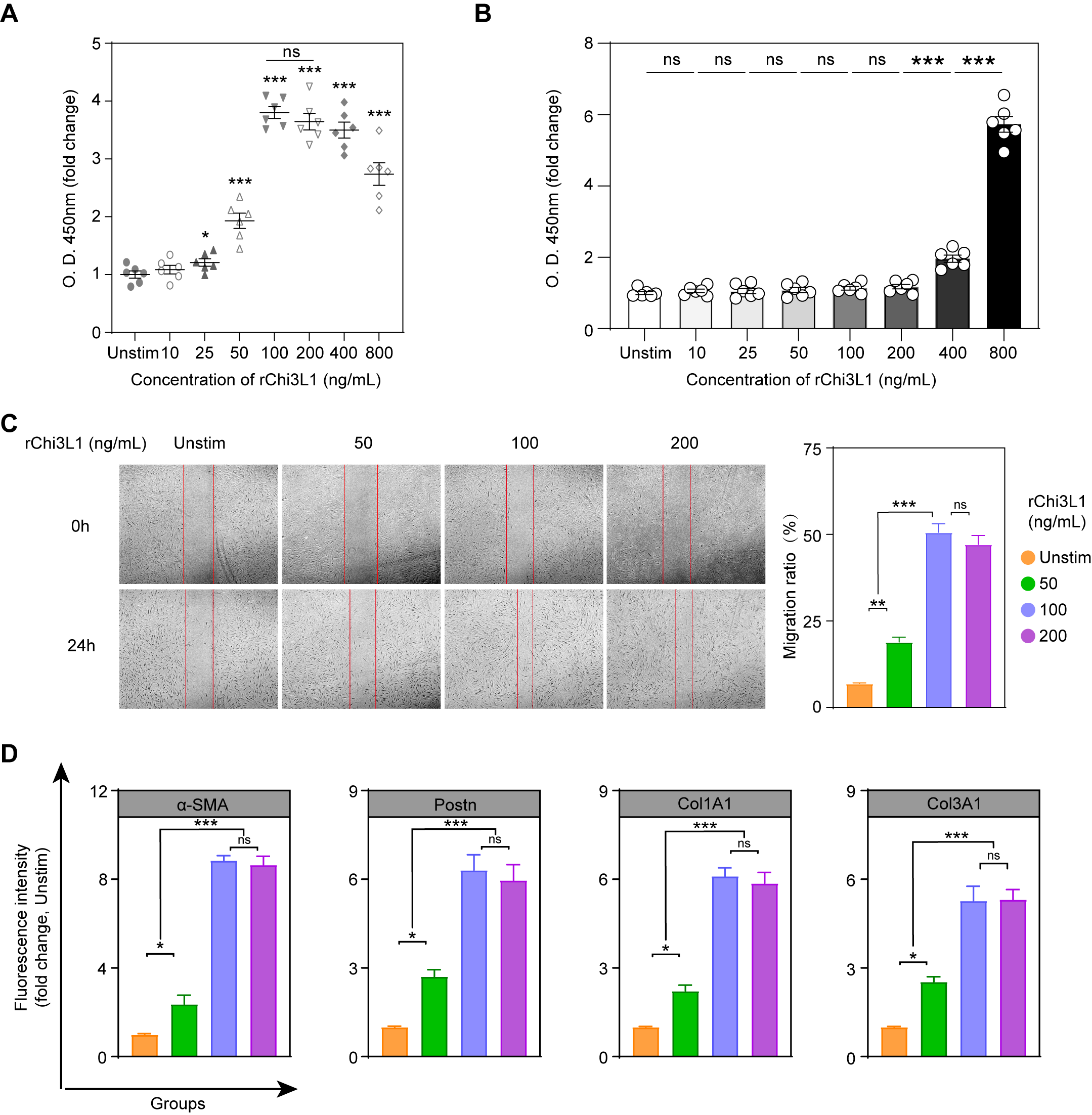


**Figure S5. A.** CCK-8 analysis used to detect SSc DFs proliferation after being stimulated with rChi3L1 for 24h. Statistical significances from the unstimulated group were evaluated. n=6/ea. **B.** The cytotoxic effect of rChi3L1 in SSc DFs for 24 h measured by the LDH assay. n=6/ea. **C.** Scratch assay used to evaluate SSc DFs migration capacities incubated with different concentrations of rChi3L1 or media alone for 24h. Magnification 40-fold. n=6/ea. **D.** Relative quantification of fluorescence intensity in SSc DFs stained with antibody against α-SMA, Postn, Col1A1 and Col3A1. n=6/ea. Data are presented as mean ± SEM. **A** and **B**, by unpaired Student’s *t*-test. **C** and **D**, by one-way ANOVA followed by Bonferroni post hoc test. ns=no significance, **P*<0.05, ***P*<0.01, ****P*<0.001 compared with corresponding groups.


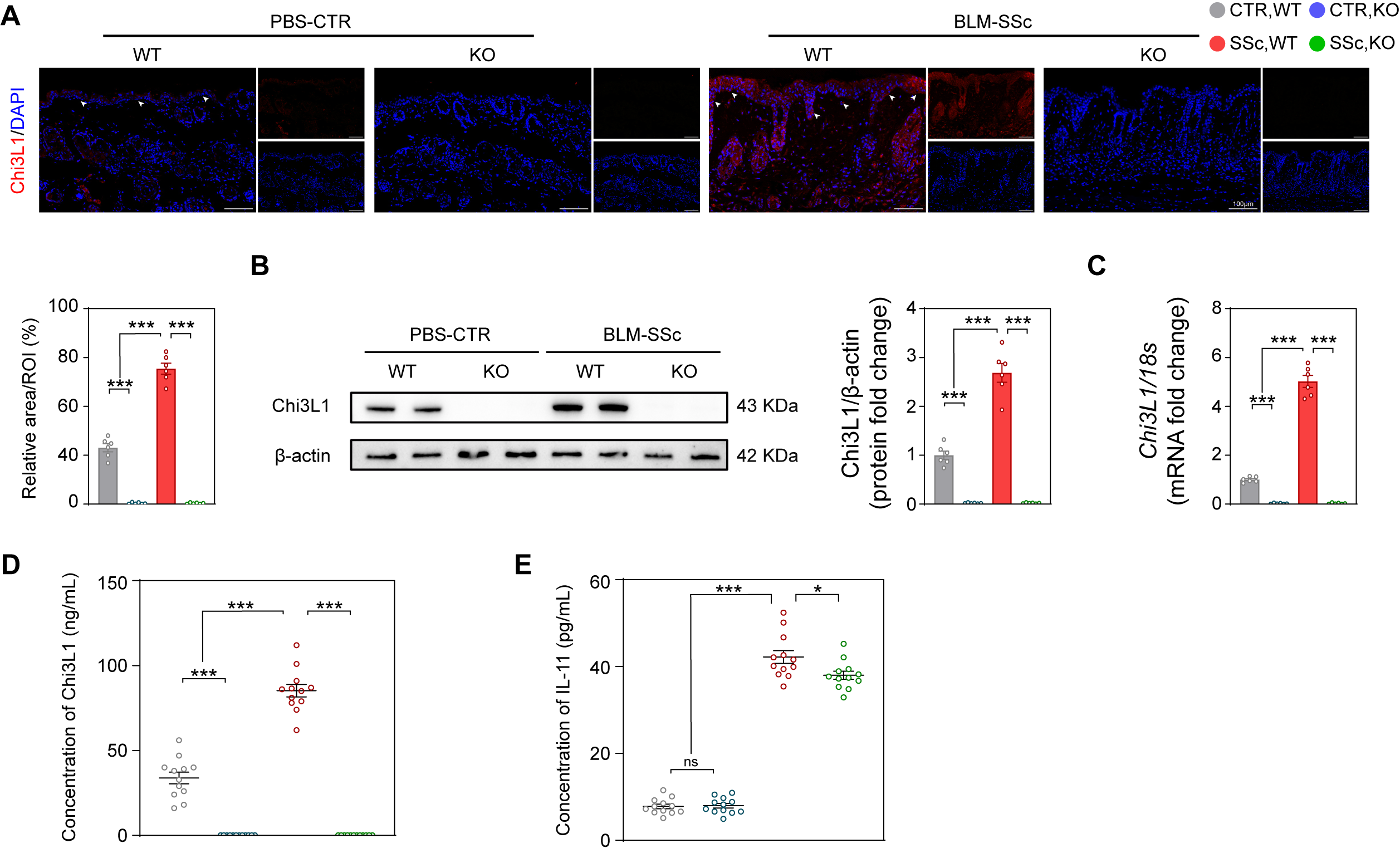


**Figure S6. A.** Representative immunofluorescent micrographs and relative quantification of Chi3L1 in WT and KO mice under PBS-CTR or BLM-SSc conditions. n=6/ea. **B.** Representative western blot and relative quantification of Chi3L1 in the corresponding groups. n=6/ea. **C.** Relative fold change of *Chi3L1* mRNA expression level in the corresponding groups. n=6/ea. **D.** Serum Chi3L1 concentration of mice in corresponding groups. n=12/ea. **E.** Serum IL-11 concentration of mice in corresponding groups n=12/ea. Data are presented as mean ± SEM. **A**, **B**, **C** and **D**, by unpaired Student’s *t*-test. **E**, by two-tailed Mann–Whitney U test. ns=no significance, **P*<0.05, ****P*<0.001 compared with corresponding groups.


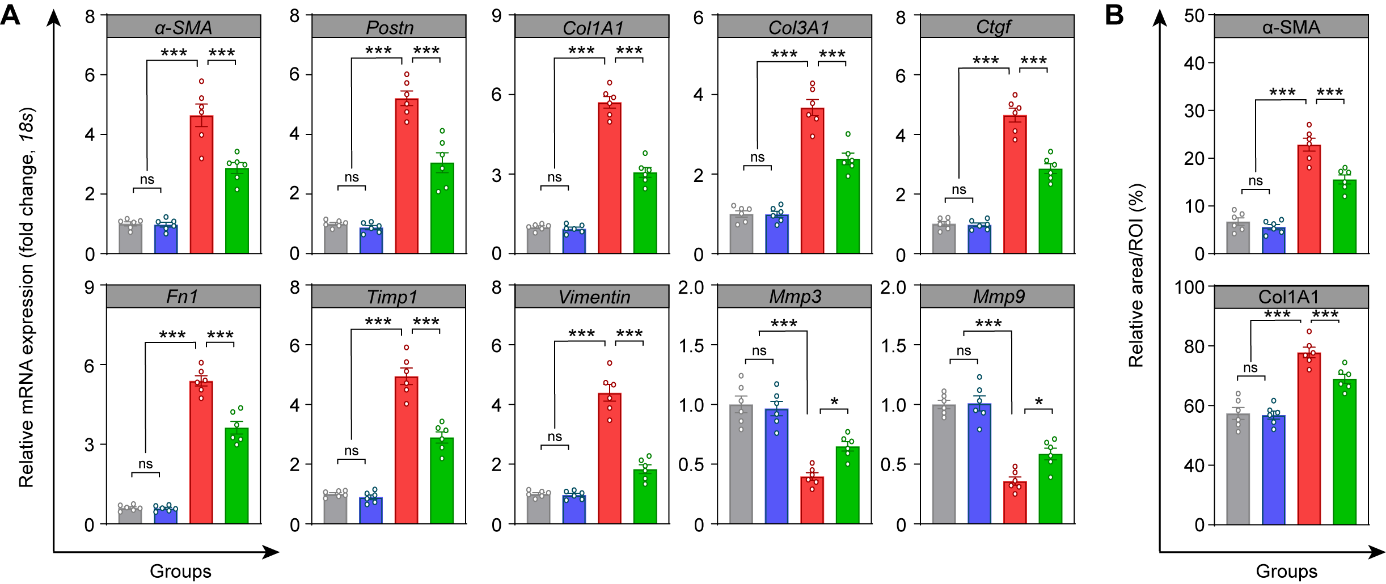


**Figure S7. A.** Relative mRNA expression of *α-SMA*, *Postn*, *Col1A1*, *Col3A1*, *Ctgf*, *Fn1*, *Timp1*, *Vimentin*, *MMP3*, and *MMP9* in each corresponding group. n=6/ea. **B.** Relative quantification of immunofluorescence stained with indicated proteins. n=6/ea. Data are presented as mean ± SEM. **A** and **B**, by one-way ANOVA followed by Bonferroni post hoc test. ns=no significance, **P*<0.05, ****P*<0.001 compared with corresponding groups.


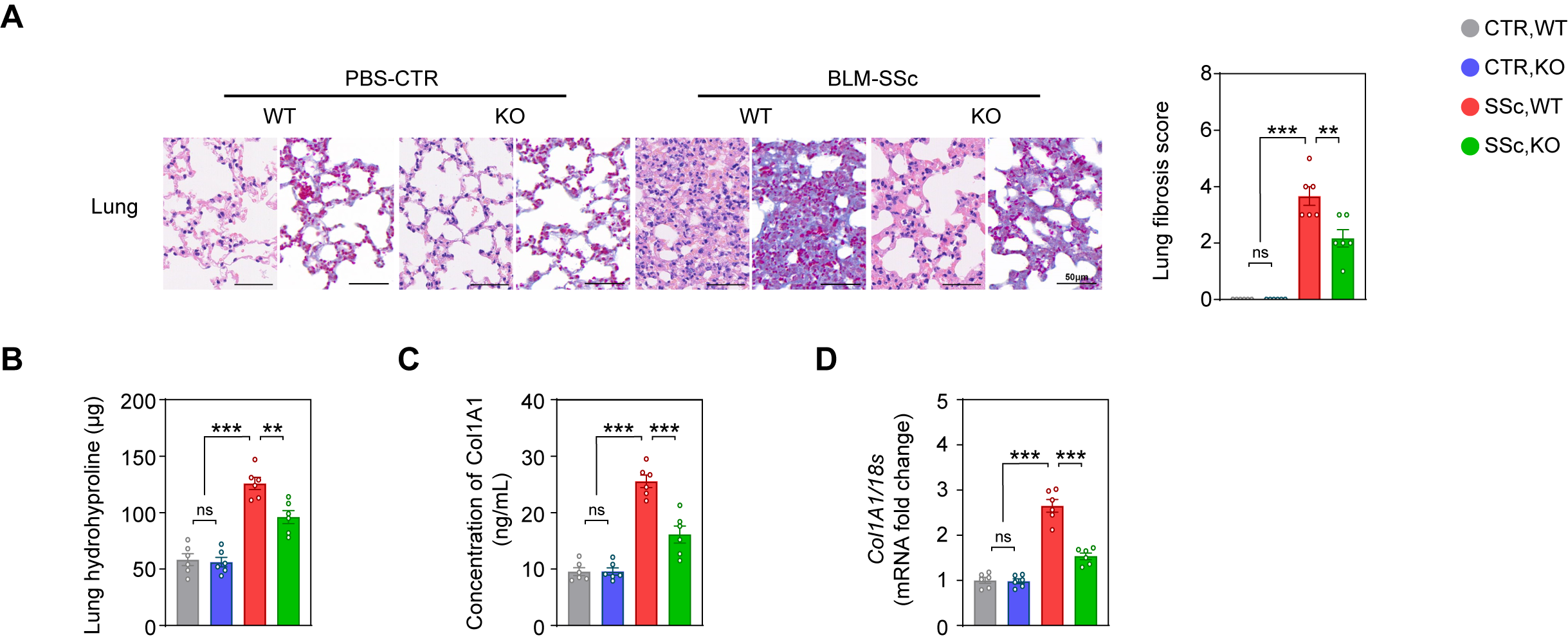


**Figure S8. A.** Representative HE (left) and Masson trichrome (right) staining of lung, and the lung fibrosis score of the corresponding groups. n=6/ea. **B.** Hydroxyproline content of lung samples in different group. n=6/ea. **C.** Protein levels of Col1A1 in the lungs. n=6/ea. **D.** Relative mRNA expression of *Col1A1* in each group. n=6/ea. Data are presented as mean ± SEM. **A**, by unpaired Student’s *t*-test. **B**, **C** and **D****,** by one-way ANOVA followed by Bonferroni post hoc test. ns=no significance, ***P*<0.01, ****P*<0.001 compared with corresponding groups.


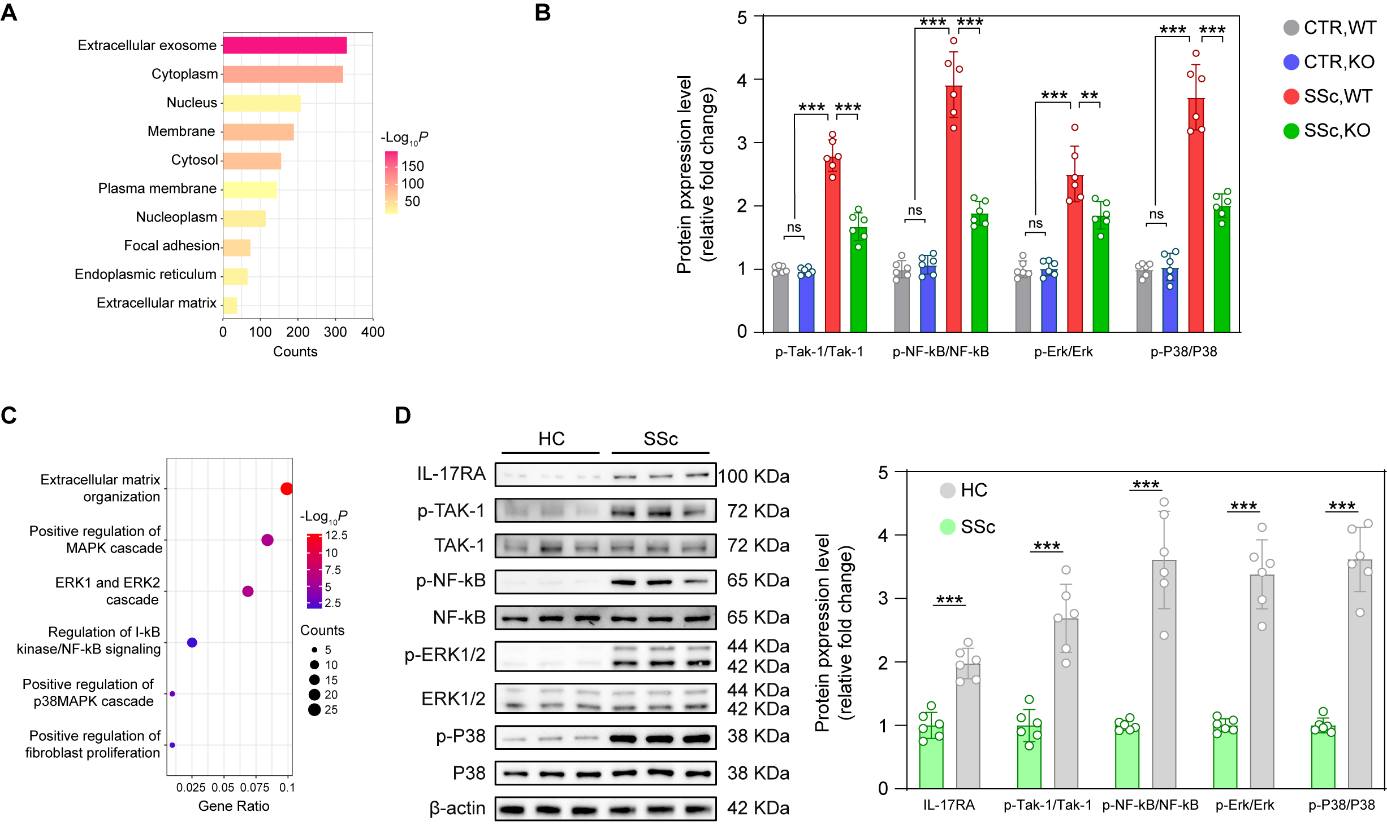


**Figure S9. A.** CC analysis of DEPs. **B.** Relative quantitative of indicated proteins in corresponding groups. n=6/ea. **C.** BP terms of DEGs of fibroblasts in scRNA-seq.  **D.** Representative WB images and relative quantitative analysis of indicated proteins in HCs and SSc groups. n=6/ea. Data are presented as mean ± SEM. **B**, by one-way ANOVA followed by Bonferroni post hoc test. **D,** by unpaired Student’s *t*-test. ns=no significance, ***P*<0.01, ****P*<0.001 compared with corresponding groups.


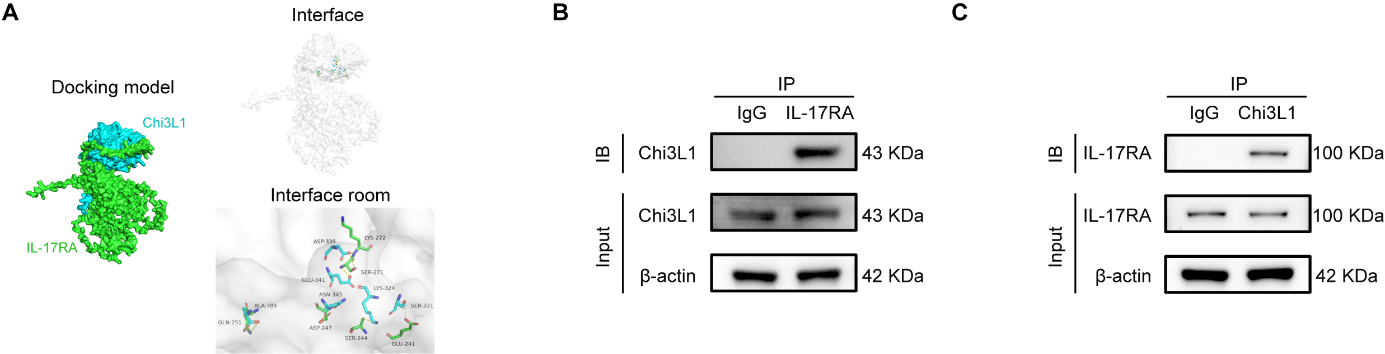


**Figure S10. A.** Surface diagram of the docking model and the interfacing residues between Chi3L1 and IL-17RA protein (Mouse source). **B, C.** Cellular CO-IP assays of Chi3L1 and IL-17RA. BLM-SSc WT mice DFs lysates were immunoprecipitated with antibody against IL-17RA, immunoblot with Chi3L1 antibody (B) and vice versa (C).


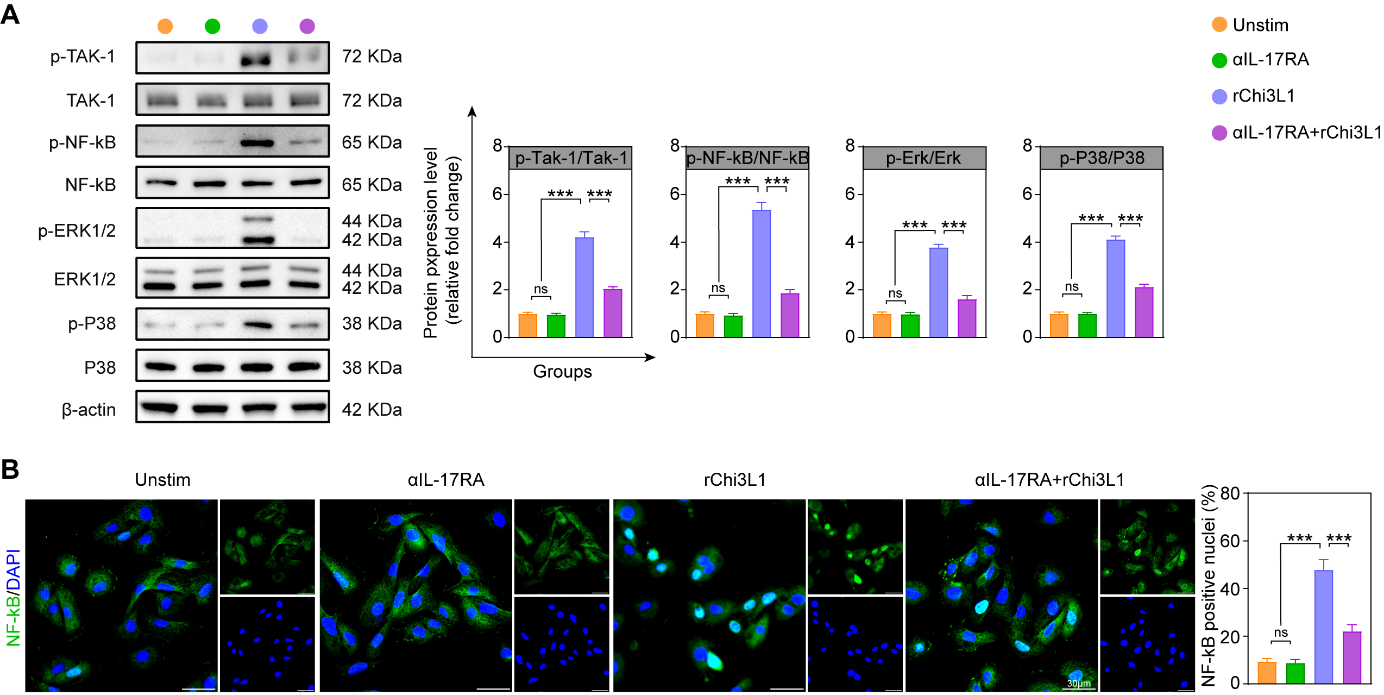


**Figure S11. Pretreatment with anti-IL-17RA mAb significantly reversed the upregulation of key phosphorylated proteins of NF-kB and P38/ERK MAPK pathways in SSc DFs induced by rChi3L1 stimulation. A.** Representative WB images and relative quantification of indicated proteins of SSc DFs after 15 min under rChi3L1 stimulation. n=6/ea. **B.** Representative immunofluorescence micrographs and relative quantification stained with NF-kB in corresponding groups. n=6/ea. Data are presented as mean ± SEM, **A** and **B**, by one-way ANOVA followed by Bonferroni post hoc test, ns=no significance, ****P*<0.001 compared with corresponding groups.


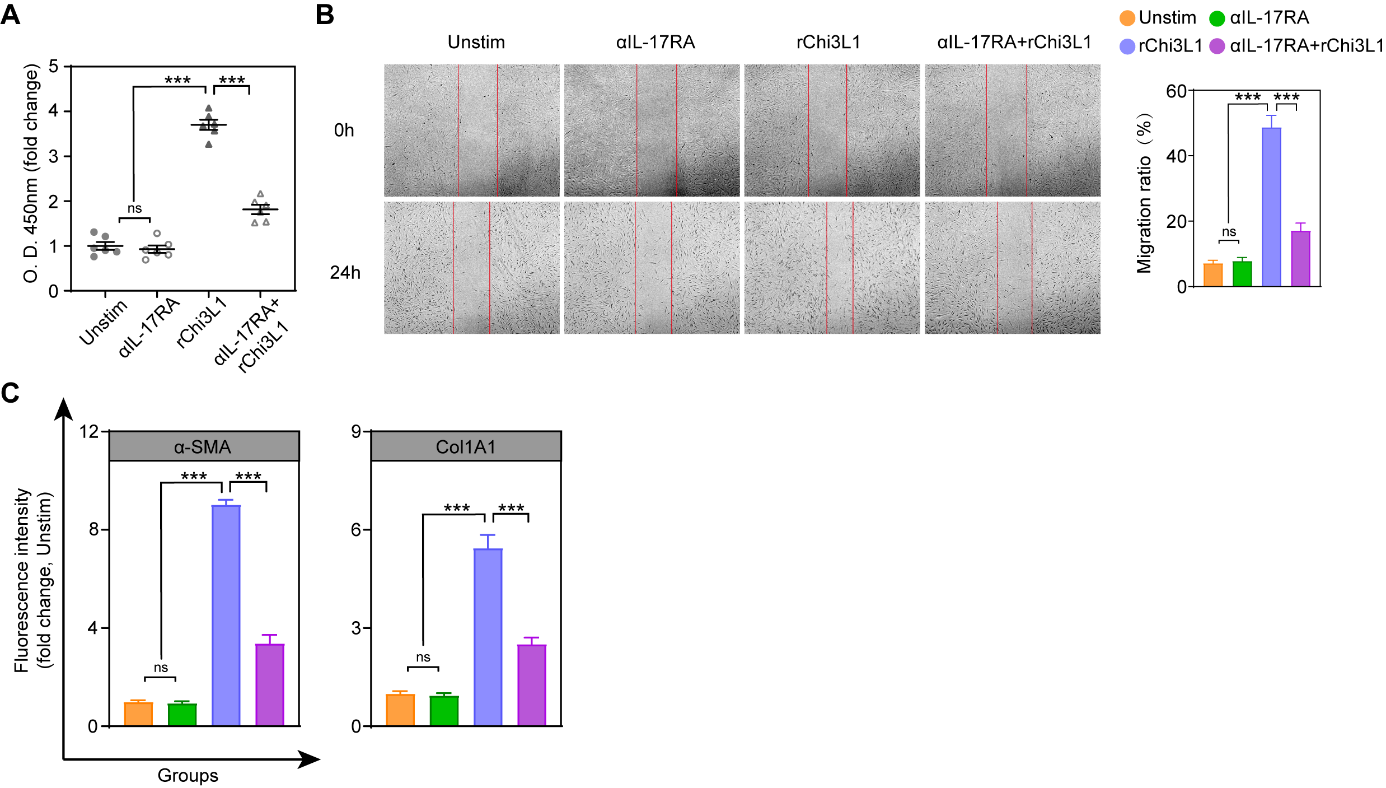


**Figure S12. A.** Proliferation measurement of SSc DFs cotreated with rChi3L1 for 24 h via CCK-8 assay. n=6/ea. **B.** Representative scratch assay images and quantification in corresponding groups with rChi3L1 administration for 24 h. n=6/ea. **C.** Relative quantification of immunofluorescence results with indicated antibodies in corresponding groups. Magnification 40-fold. n=6/ea. Data are presented as mean ± SEM, **A**, **B**, and **C**, by one-way ANOVA followed by Bonferroni post hoc test, ns=no significance, ****P*<0.001 compared with corresponding groups.


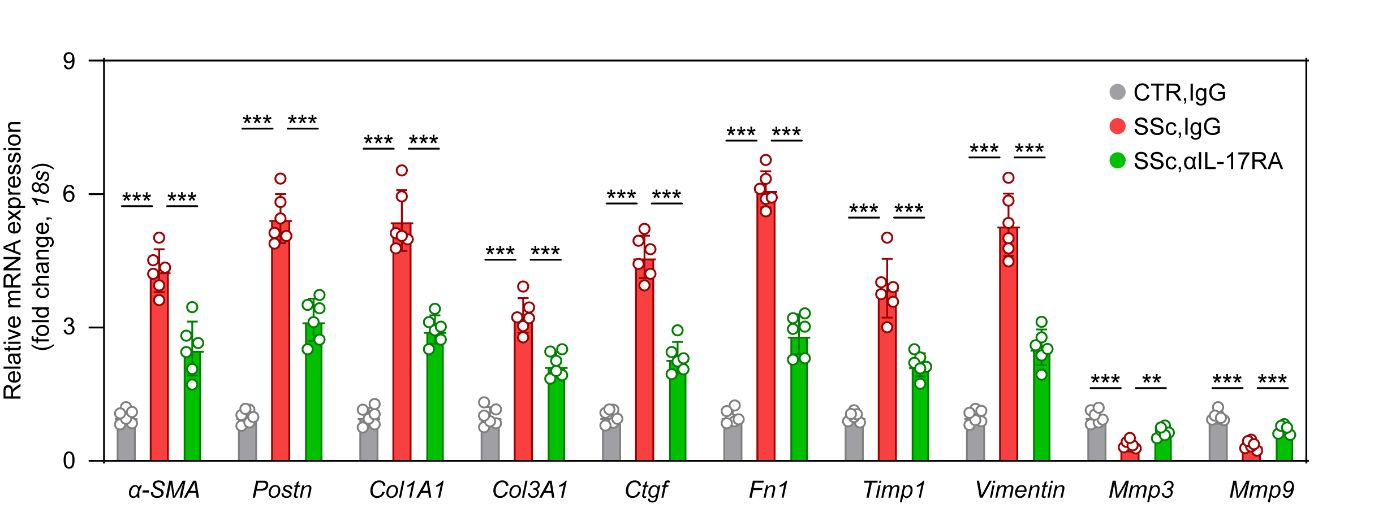


**Figure S13.** Relative mRNA expression of *α-SMA*, *Postn*, *Col1A1*, *Col3A1*, *Ctgf*, *Fn1*, *Timp1*, *Vimentin*, *MMP3*, and *MMP9* in each corresponding group. n=6/ea. Data are presented as mean ± SEM. By one-way ANOVA followed by Bonferroni post hoc test. ns=no significance, ***P*<0.01, ****P*<0.001 compared with corresponding groups.


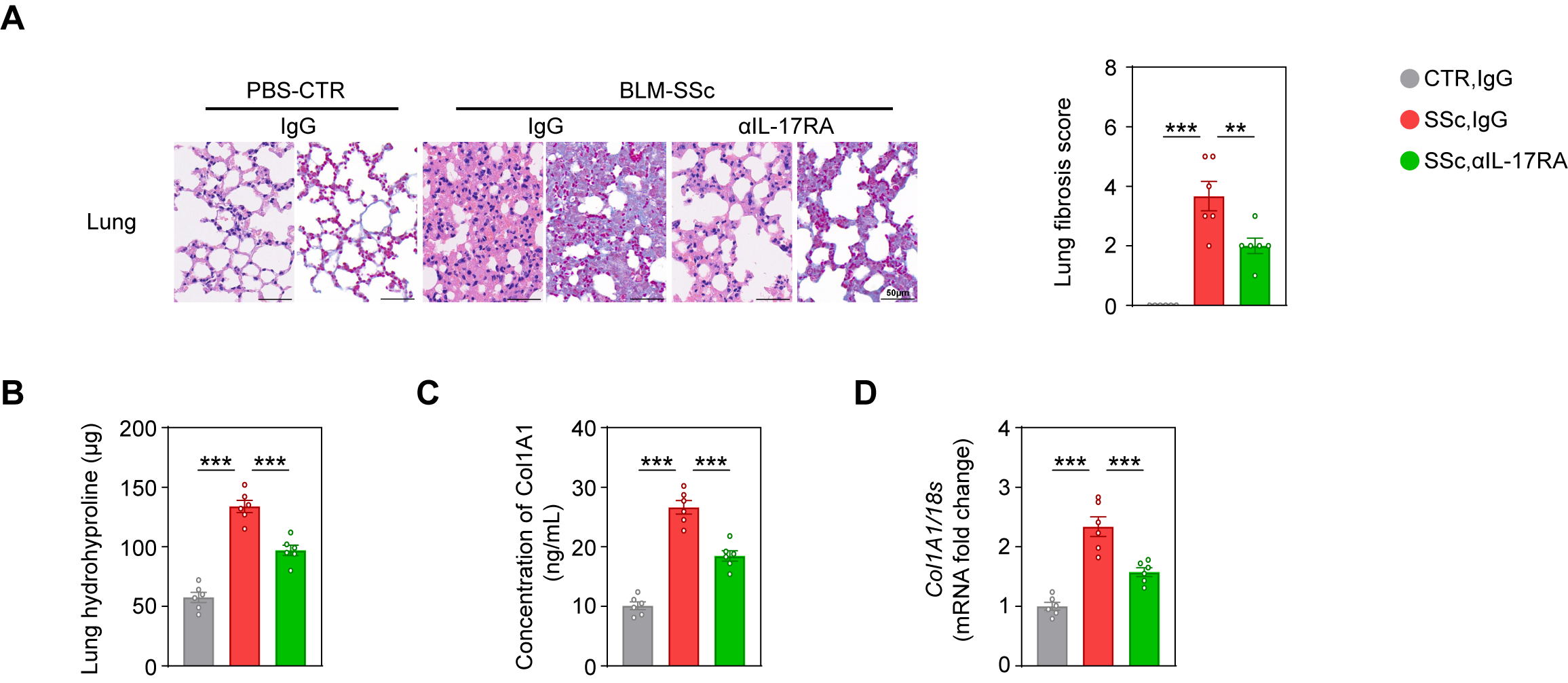


**Figure S14.** **A.** Representative HE staining (left) and Masson trichrome (right) of lung, and the lung fibrosis score of corresponding groups. n=6/ea. **B.** Hydroxyproline contents of lung samples. n=6/ea. **C.** Protein levels of Col1A1 in the lungs. n=6/ea. **D.** Relative mRNA expression of *Col1A1* in each group. n=6/ea. Data are presented as mean ± SEM. **A**, by unpaired Student’s *t*-test. **B**, **C** and **D**, by one-way ANOVA followed by Bonferroni post hoc test. ns=no significance, ***P*<0.01, ****P*<0.001 compared with corresponding groups.

**Supplemental Tables**

**Table S1** **Clinical features of 72 participants**

| **Characteristics** | **SSc (n=48)** | | **HCs (n=24)** |  |
| --- | --- | --- | --- | --- |
|  | **dcSSc (n=22)** | **lcSSc (n=26)** |  |  |
| Age (at onset), median (quartiles) years | 44 (36-53) | 49 (39-58) | 48 (38-55) |  |
| Sex, n (%) |  |  |  |  |
| Male | 3 (14) | 5 (19) | 4 (17) |  |
| Female | 19 (86) | 21 (81) | 20 (83) |  |
| Disease duration, median (quartiles) years | 1 (0-1) | 1 (0-2) | / |  |
| MRSS, median (quartiles) points | 12 (17-25) **^∗∗∗^** | 10 (6-12) | / |  |
| Pulmonary involvement |  |  |  |  |
| ILD, n% | 12 (55) | 11 (42) | / |  |
| %VC, median (quartiles) % | 83 (70-91) | 87 (80-93) | / |  |
| %DLCO, median (quartiles) % | 78 (61-85) | 81 (75-88) | / |  |
| Chi3L1, median (quartiles) ng mL^-1^ | 99 (92-121) **^∗^** | 90 (83-104) | 39 (33-46) ^###^ |  |

SSc: systemic sclerosis; dcSSc: diffuse cutaneous systemic sclerosis; lcSSc: limited cutaneous systemic sclerosis; HC: healthy control; MRSS: Modified Rodnan skin score; ILD: Interstitial lung disease; VC: vital capacity; DLCO: diffusing capacity for carbon monoxide. Significance was determined by Fisher’s exact probability test for comparison of proportions and two-tailed Mann–Whitney U test for quantitative variables. **P*<0.05, ****P*<0.001 vs patients with lcSSc; ^###^*P*<0.001 vs patients with SSc.

**Table S2 Results of molecular docking (Human)**

| Receptor | Ligand | Binding energy | Interface area (Å²) | Hydrogen bonds (Chi3L1: IL-17RA) |
| --- | --- | --- | --- | --- |
| IL-17RA | Chi3L1 | -28.2kcal mol^-1^ | 2482.4 | HIS 149: GLU 241 |
|  |  |  |  | ASP 199: SER 329 |
|  |  |  |  | LEU 198: SER 329 |
|  |  |  |  | SER 279: ARG 452 |
|  |  |  |  | ARG 286: ARG 568 |
|  |  |  |  | LYS 23: SER 329 |
|  |  |  |  | LYS 289: ASP 556 |

**Table S3 Results of the interaction between Chi3L1 and IL-17RA determined by SPR**

| Ligand | Determinand | K_D_ (M) | Ka (Ms^-1^) | Kd (s^-1^) |
| --- | --- | --- | --- | --- |
| IL-17RA (Human) | Chi3L1 (Human) | 2.96 × 10^-7^ | 1.35 × 10^5^ | 4.00 × 10^-2^ |

K_D_: dissociation constant; Ka: association rate constant; Kd: dissociation rate constant.

**Table S4 Results of molecular docking (Mouse)**

| Receptor | Ligand | Binding energy | Interface area (Å²) | Hydrogen bonds (Chi3L1: IL-17RA) |
| --- | --- | --- | --- | --- |
| IL-17RA | Chi3L1 | -27.7kcal mol^-1^ | 3245.7 | GLN 223: ILE 327 |
|  |  |  |  | GLU 280: ILE 319 |
|  |  |  |  | GLU 280: TYR 318 |
|  |  |  |  | ASP 339: SER 271 |
|  |  |  |  | ASP 339: LYS 272 |
|  |  |  |  | GLU 341: CYS 276 |
|  |  |  |  | ALA 389: GLN 251 |
|  |  |  |  | ARG 8: SER 55 |
|  |  |  |  | ARG 8: CYS 126 |
|  |  |  |  | ASN 345: ASP 247 |
|  |  |  |  | ASN 281: ASP 317 |
|  |  |  |  | GLN 282: ASP 317 |
|  |  |  |  | ARG 130: ILE 363 |
|  |  |  |  | GLN 259: GLU 534 |
|  |  |  |  | ASN 167: MET 550 |

**Table S5 Study subjects of scRNA-seq**

|  | SSc n=3 | HCs n=3 |
| --- | --- | --- |
| Age, median (range) | 41 (32-49) | 37 (29-45) |
| Sex, n% |  |  |
| Male | 1 (33.3) | 1 (33.3) |
| Female | 2 (66.6) | 2 (66.6) |
| Ethnicity, n% | Asian (100) | Asian (100) |
| Disease duration, median (range) years | 1(0-2) | / |
| Skin involvement, n% |  |  |
| Limited | 1(33.3) | / |
| Diffuse | 2(66.6) | / |

**Table S6 DFs from patients with SSc used for *in vitro* experiments**

|  | SSc n=6 |
| --- | --- |
| Age, median (range) | 37 (24-52) |
| Sex, n% |  |
| Male | 1 (16.7) |
| Female | 5 (83.3) |
| Ethnicity, n% | Asian (100) |
| Disease duration, median (range) years | 1(0-2) |
| Skin involvement, n% |  |
| Limited | 0(0) |
| Diffuse | 6(100) |

**Table S7 Primers for qPCR analysis**

| Gene | Forward Primer (5’ to 3’) | Reverse Primer (5’ to 3’) |
| --- | --- | --- |
| Human | | |
| *Chi3L1* | TGGGTCTCAAAGATTTTCCAAGA | GCCTCAACATGTACCCCACA |
| *α-SMA* | TCTGGAGATGGTGTCACCCACAAT | AATAGCCACGCTCAGTCAGG |
| *Postn* | CAACGCAGCGCTATTCTGAC | TCGGAAGCCACTTTGTCTCC |
| *Col1A1* | CCAGAAGAACTGGTACATCAGCA | CGCCATACTCGAACTGGGAAT |
| *Col3A1* | AATCAGGTAGACCCGGACGA | CTCCTGGGATGCCATTTGGT |
| *Fn1* | AAGAAGGGCTCGTGTGACAG | TCTTGTCCTACATTCGGCGG |
| *Timp1* | TTGGCTGTGAGGAATGCACA | GTCCACAAGCAATGAGTGCC |
| *18s* | GGAGTATGGTTGCAAAGCTGA | ATCTGTCAATCCTGTCCGTGT |
| Mouse | | |
| *Chi1L3* | AGTCTGGCGCCAAATCACAG | CTGGGCTCCCAGACGTATCA |
| *α-SMA* | GTCCCAGACATCAGGGAGTAA | TCGGATACTTCAGCGTCAGGA |
| *Postn* | CGGGAAGAACGAATCATTACA | ACCTTGGAGACCTCTTTTTGC |
| *Col1A1* | TCGTGGCTTCTCTGGTCTC | CCGTTGAGTCCGTCTTTGC |
| *Col3A1* | CTGTAACATGGAAACTGGGGAAA | CCATAGCTGAACTGAAAACCACC |
| *Ctgf* | GTGCCAGAACGCACACTG | CCCCGGTTACACTCCAAA |
| *Fn1* | AAGAGGACGTTGCAGAGCTA | AGACACTGGAGACACTGACTAA |
| *Timp1* | GCATCTGGCATCCTCTTGTTG | GGTGGTCTCGTTGATTTCTGG |
| *Vimentin* | ACTAGCCGCAGCCTCTATTCCTC | GAAGTCCACCGAGTCTTGAAGCAG |
| *Mmp3* | GGCTGTGTGTGGTTGTGTGCTC | CCTCCTCCCAGACCTTCAAAGC |
| *Mmp9* | AGACGACATAGACGGCATCC | TGGGACACATAGTGGGAGGT |
| *18s* | ACCGCAGCTAGGAATAATGGA | CAAATGCTTTCGCTCTGGTC |

**Table S8 Antibodies used in this study**

| **Antibody** | **Company** | **Cat. No.** | **Application** | **Dilution** |
| --- | --- | --- | --- | --- |
| Chi3L1 | Invitrogen | MA5-36122 | WB | 1:1000 |
| Chi3L1 | Invitrogen | MA5-36122 | IF | 1:200 |
| Chi3L1 | Invitrogen | MA5-36122 | IP | 1:100 |
| CALML3 | Invitrogen | PA5-30232 | IF | 1:200 |
| α-SMA | Abcam | ab7817 | WB | 1:1000 |
| α-SMA | Abcam | ab7817 | IF | 1:200 |
| Postn | Abcam | ab92460 | WB | 1:1000 |
| Postn | Abcam | ab92460 | IF | 1:200 |
| Col1A1 | Abcam | ab34710 | WB | 1:1000 |
| Col1A1 | Abcam | ab34710 | IF | 1:200 |
| Col3A1 | Abcam | ab7778 | WB | 1:1000 |
| Col3A1 | Abcam | ab7778 | IF | 1:200 |
| IL-17RA | ABclonal | A10052 | WB | 1:1000 |
| IL-17RA | ABclonal | A10052 | IF | 1:200 |
| IL-17RA | ABclonal | A10052 | IP | 1:50 |
| TAK-1 | Abcam | ab109526 | WB | 1:1000 |
| p-TAK-1 | Abcam | ab109404 | WB | 1:1000 |
| P38 | Abcam | ab170099 | WB | 1:1000 |
| p-P38 | Abcam | ab178867 | WB | 1:1000 |
| ERK1/2 | Abcam | ab17942 | WB | 1:1000 |
| p-ERK1/2 | Abcam | ab278538 | WB | 1:1000 |
| NF-kB | Proteintech | 10745-1-AP | WB | 1:1000 |
| NF-kB | Proteintech | 10745-1-AP | IF | 1:200 |
| p-NF-kB | Proteintech | 82335-1-RR | WB | 1:1000 |
| Ki67 | Abcam | ab15580 | IF | 1:200 |
| β-actin | Proteintech | HRP-60008 | WB | 1:20000 |
